# High‐dimensionality undersampled patch‐based reconstruction (HD‐PROST) for accelerated multi‐contrast MRI

**DOI:** 10.1002/mrm.27694

**Published:** 2019-03-04

**Authors:** Aurélien Bustin, Gastão Lima da Cruz, Olivier Jaubert, Karina Lopez, René M. Botnar, Claudia Prieto

**Affiliations:** ^1^ Department of Biomedical Engineering, School of Imaging Sciences & Biomedical Engineering King’s College London, King’s Health Partners London United Kingdom; ^2^ Escuela de Ingeniería Pontificia Universidad Católica de Chile Santiago Chile

**Keywords:** compressed‐sensing, low‐rank tensor decomposition, magnetization transfer contrast, MR fingerprinting, multi‐contrast MRI, patch‐based reconstruction

## Abstract

**Purpose:**

To develop a new high‐dimensionality undersampled patch‐based reconstruction (HD‐PROST) for highly accelerated 2D and 3D multi‐contrast MRI.

**Methods:**

HD‐PROST jointly reconstructs multi‐contrast MR images by exploiting the highly redundant information, on a local and non‐local scale, and the strong correlation shared between the multiple contrast images. This is achieved by enforcing multi‐dimensional low‐rank in the undersampled images. 2D magnetic resonance fingerprinting (MRF) phantom and in vivo brain acquisitions were performed to evaluate the performance of HD‐PROST for highly accelerated simultaneous T_1_ and T_2_ mapping. Additional in vivo experiments for reconstructing multiple undersampled 3D magnetization transfer (MT)‐weighted images were conducted to illustrate the impact of HD‐PROST for high‐resolution multi‐contrast 3D imaging.

**Results:**

In the 2D MRF phantom study, HD‐PROST provided accurate and precise estimation of the T_1_ and T_2_ values in comparison to gold standard spin echo acquisitions. HD‐PROST achieved good quality maps for the in vivo 2D MRF experiments in comparison to conventional low‐rank inversion reconstruction. T_1_ and T_2_ values of white matter and gray matter were in good agreement with those reported in the literature for MRF acquisitions with reduced number of time point images (500 time point images, ~2.5 s scan time). For in vivo MT‐weighted 3D acquisitions (6 different contrasts), HD‐PROST achieved similar image quality than the fully sampled reference image for an undersampling factor of 6.5‐fold.

**Conclusion:**

HD‐PROST enables multi‐contrast 2D and 3D MR images in a short acquisition time without compromising image quality. Ultimately, this technique may increase the potential of conventional parameter mapping.

## 1 INTRODUCTION

In MRI, multiple contrasts are exploited to extract clinically relevant tissue parameters and pathological tissue changes. These multiple contrasts are achieved using different imaging sequences and preparation pulses. Multi‐contrast acquisitions also find important applications in parameter mapping (e.g., T_1_ and T_2_ mapping) and magnetic resonance fingerprinting (MRF).^1^, ^2^ However, these acquisitions lead to long scan times because multiple images with different contrasts need to be acquired, making parameter imaging more sensitive to physiological motion.^3^, ^4^, ^5^, ^6^

Parallel imaging (PI),^7^, ^8^, ^9^, ^10^, ^11^ compressed sensing (CS),^12^, ^13^ as well as the combination of both undersampled reconstruction techniques^14^, ^15^ have been proposed to overcome the long scan times associated with multi‐contrast imaging and parameter mapping. PI can accelerate multi‐contrast imaging by undersampling each individual image and exploiting the information provided by multiple coil arrays, yet at a SNR penalty generally marked for high acceleration factors. Sparse CS alone has been shown to cope with the problem of undersampling through the use of random or pseudo‐random sampling patterns and efficient regularized reconstructions that make the assumption that the multi‐contrast images share common and sparse information in a specific domain.^16^, ^17^, ^18^, ^19^, ^20^, ^21^ Even though these strategies have achieved acceleration factors that have not previously been possible to attain with parallel imaging alone, CS‐based techniques still suffer from residual aliasing artifacts for high acceleration factors, which compromise the diagnostic value of the reconstructed multi‐contrast images.

Recently, novel techniques that exploit the strong anatomical correlations observed in the contrast dimension (or parameter dimension) on a global or local scale have been proposed. Indeed, the nature of signal evolution in multi‐contrast acquisitions exhibits a low‐rank structure in the contrast dimension that can be exploited to further reduce scan times.^17^, ^22^, ^23^, ^24^ These types of reconstruction techniques, also known as the globally (GLR) or locally low‐rank (LLR) methods,^25^ have been efficiently used in many applications such as T_2_ mapping^26^ or dynamic contrast enhanced MRI.^27^ More recently, high‐order tensor decomposition techniques, exploiting global correlation, have been efficiently used to allow for highly accelerated multi‐dimensional cardiac MRI acquisitions.^28^, ^29^ Although those techniques have shown promise for motion‐resolved quantitative cardiac imaging by efficiently solving a global low‐rank tensor decomposition, they do not exploit the strong non‐local correlations between neighboring patches.

Motivated by the LLR techniques that exploit localized correlations in the contrast dimension, patch‐based image reconstructions exploiting non‐local spatial redundancies and low‐rank matrix structures have been introduced for single‐contrast MRI reconstruction to lead to even sparser representation.^30^, ^31^ By modeling the similarity of image patches through block‐matching, low‐rank representation and filtering, 2D,^32^ and 3D^33^ patch‐based reconstructions have been shown to outperform conventional CS reconstructions by recovering better image details and edges and exhibiting better overall image quality.

In this study, we present a new reconstruction technique for highly accelerated 2D and 3D multi‐channel multi‐contrast MRI that combines the promising performances of patch‐based reconstructions and the potential of low‐rank image reconstruction through higher‐order tensor decomposition. The proposed high‐dimensionality undersampled patch‐based reconstruction (HD‐PROST) technique is first applied to accelerated 2D radial MRF, for various acceleration factors, where a high degree of inherent redundancy can be exploited locally, non‐locally, and through the contrast dimension. In a second application, HD‐PROST is used to acquire multiple undersampled high‐resolution 3D Cartesian magnetization transfer contrast (MTC) images with several MT weightings in a reduced scan time.

## 2 THEORY

The framework presented hereafter jointly reconstructs multi‐channel multi‐contrast images from undersampled 2D or 3D MR acquisitions. This is achieved by generalizing our previously proposed PROST technique^33^ to high dimensional imaging. A description of the proposed HD‐PROST reconstruction is presented, followed by the description of 2 multi‐contrast applications (2D radial and 3D Cartesian) where high‐dimensionality can be exploited to reduce acquisition time, which is often a key factor for clinical translation.

### 2.1 High‐dimensionality undersampled patch‐based reconstruction (HD‐PROST)

Let $X\in C^{M_{x}\;\times \;M_{y}\;\times \;M_{z}\;\times \;L}$ be the multi‐contrast complex images that we seek to reconstruct, where $M_{x}$, $M_{y}$ and $M_{z}$ are the number of voxels in the x, y and z spatial directions, and L is the number of contrast‐weighted images. The corresponding complex receive‐coil sensitivity maps for the $N_{c}$ channels are denoted as $S\in C^{M_{x}\;\times \;M_{y}\;\times \;M_{z}\;\times \;N_{c}}$. Let $Y\in C^{Z\;\times \;L\;\times \;N_{c}}$ be the undersampled k‐space data (with $Z\ll M_{x}\;\times \;M_{y}\;\times \;M_{z}$). The joint multi‐contrast undersampled reconstruction can be combined with parallel imaging and cast as the following inverse problem

(1) $$\underset{X}{\text{argmin}}\frac{1}{2}{\left\|AFSX-Y\right\|}_{F}^{2}$$

where A is the undersampling operator that acquires k‐space data for each contrast‐weighted image, F denotes the Fourier transform operator and ${\left\|\;\cdot \;\right\|}_{F}$ is the Frobenius norm. Mathematically, this inverse problem is ill‐posed, in the sense that the exact solution might not exist or not be unique, making precise recovery of X hardly possible, and prior assumptions on the unknown solution X have to be considered.

The principle behind HD‐PROST reconstruction assumes that a multi‐contrast image X can be expressed as a high‐order low‐rank representation on a patch scale, with respect to an appropriately chosen patch selection operator. The recovery problem can be formulated as the following constrained optimization on the high‐order low‐rank tensor T

(2) $$\underset{X}{\text{argmin}}\frac{1}{2}{\left\|AFSX-Y\right\|}_{F}^{2}+\sum_{p}\lambda_{p}{\left\|T_{p}\right\|}_{*}\;s.t.\;T_{p}=P_{p}\left(X\right)$$

where $\lambda_{p}$ is the nonnegative sparsity‐promoting regularization parameter and ${\left\|\;\cdot \;\right\|}_{*}$ is the nuclear norm that enforces multi‐dimensional low‐rank on a multi‐contrast patch scale. The patch selection operator $P_{p}\left(\cdot \right)$ forms a 3D tensor from a patch centered at pixel *p* from a set of multi‐contrast images (see optimization 2 below). Now considering the constraint $T_{p}=P_{p}\left(X\right)$, and the encoding operator E=AFS, we can form the unconstrained Lagrangian of Equation 2 by linearly combining the constraint and cost function^31^, ^33^

(3) $$L_{HD-PROST}\left(X,T,b\right)\;:=\underset{X,T,b}{\text{argmin}}\frac{1}{2}{\left\|EX-Y\right\|}_{F}^{2}+\;\sum_{p}\lambda_{p}{\left\|T_{p}\right\|}_{*}+\frac{\mu }{2}\sum_{p}\|T_{p}-P_{p}\left(X\right)-\frac{b_{p}}{\mu }{\|}_{F}^{2}$$

where b is the Lagrange multiplier, and μ>0 is the penalty parameter. Equation 3 can be efficiently solved through operator‐splitting via alternating direction method of multipliers (ADMM).^34^ ADMM simplifies the optimization process by alternating the minimization with respect to the multi‐contrast set of images X (optimization 1) and the high‐order tensor T (optimization 2) followed by an update of the augmented multiplier b, and repeating these 3 steps until a convergence criterion is satisfied.

#### 2.1.1 Optimization 1: joint MR reconstruction update

The first sub‐problem is a joint multi‐contrast MR reconstruction that incorporates the denoised tensor T (obtained at the end of optimization 2) as prior information in a parallel imaging fashion to obtain X

(4) $$L_{\mathrm{JointRecon}}\left(X\right)\;:=\underset{X}{\text{argmin}}\frac{1}{2}{\left\|EX-Y\right\|}_{F}^{2}+\frac{\mu }{2}{\left\|T-X-\frac{b}{\mu }\right\|}_{F}^{2}$$

Equation 4 corresponds to a standard iterative SENSE reconstruction with Tikhonov regularization, where the solution X can be efficiently computed using the Conjugate Gradient^35^ algorithm.

#### 2.1.2 Optimization 2: high order singular value decomposition (HOSVD)‐based denoising

Considering the variable ${\tilde{T}}_{p}=P_{p}\left(X\right)+\frac{b_{p}}{\mu }$, the second sub‐problem minimizes with respect to the high‐order tensor T and is given by

(5) $$L_{\mathrm{Tensor}}\left(T\right)\;:=\underset{T}{\text{argmin}}\sum_{p}\frac{2\lambda_{p}}{\mu }\|T_{p}{\|}_{*}+\sum_{p}{\left\|T_{p}-{\tilde{T}}_{p}\right\|}_{F}^{2}$$

**Figure 1:**
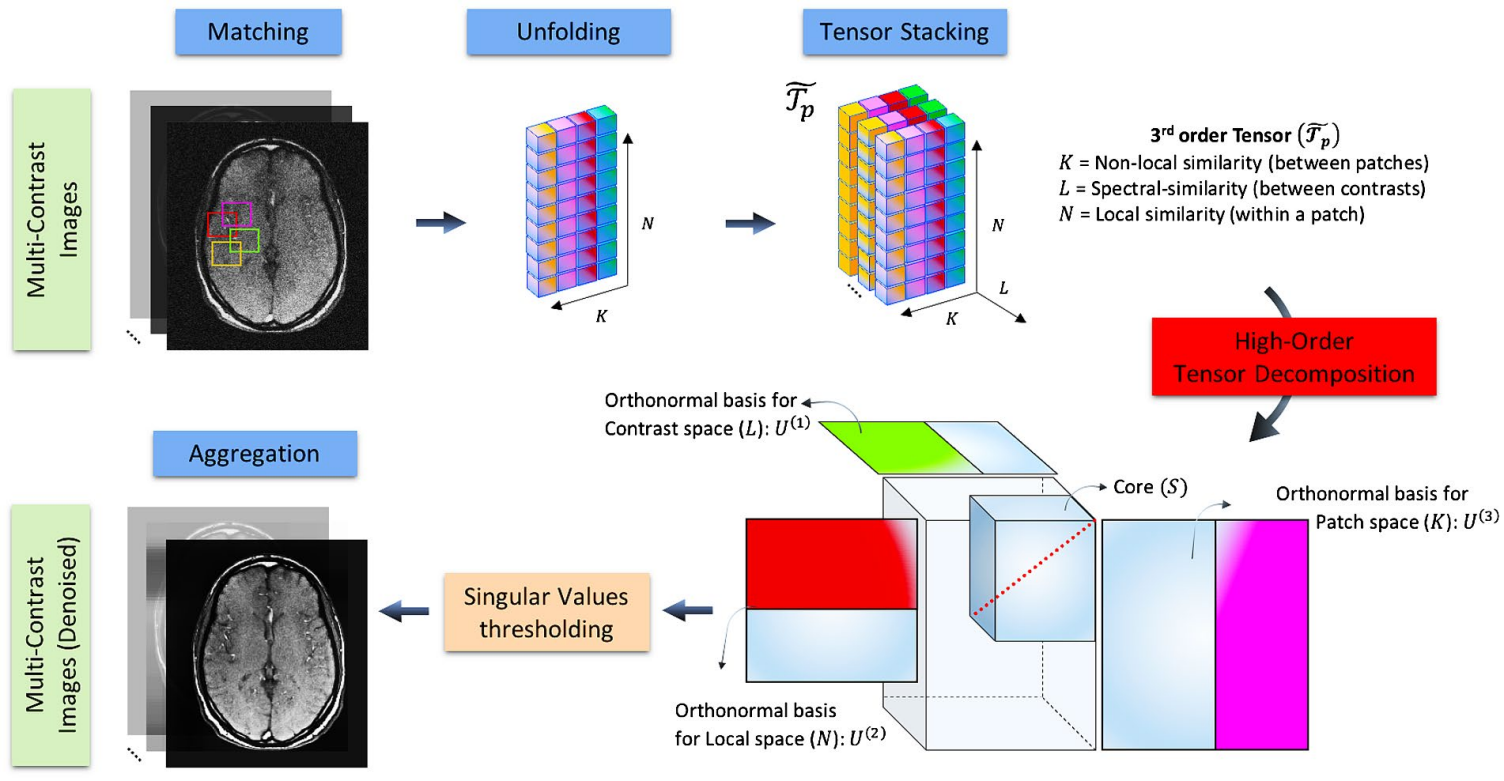
Flowchart of the optimization 2 of the proposed high‐dimensionality patch‐based reconstruction (HD‐PROST). Denoising of multi‐contrast images is performed using 2D (respectively 3D) block matching, which groups similar 2D (respectively 3D) patches in the multi‐contrast images. Similar patches are then unfolded together in a simple 2D matrix. A third‐order tensor ${\tilde{T}}_{p}$ is formed by stacking the unfolded patches in the contrast dimension. The high‐order tensor of size N×K×L admits a low multilinear rank approximation and can be compressed, through tensor decomposition, by truncating the multilinear singular vectors that correspond to small multilinear singular values. The outputs of this step are the denoised multi‐contrast images that are then used in the joint MR reconstruction process (optimization 1) as prior knowledge. An overview of the algorithm is provided in Supporting Information Table S1

X denotes multiple MR images with different contrasts. Several observations can be made about X: (1) on a local scale, voxels at a specific location for a given contrast exhibit similar intensity to their nearest neighbors (within a patch), (2) on a non‐local scale, images for a given contrast contain self‐repeating patterns (measured as patch similarity within a neighborhood), and (3) on a contrast scale, common structures and features are shared across multiple contrast images. Motivated by these observations, the proposed joint multi‐channel multi‐contrast problem can be cast as a multi‐dimensional low‐rank reconstruction. Bearing this in mind, equation 5 can be solved on a multi‐contrast patch level. The construction of the high‐order tensor T is performed as a 3‐step process:

Step 1 **–** Similar overlapping patches in $X+\frac{b}{\mu }$ are grouped together to form a third‐order tensor: considering a 3D+L reference patch of size $N_{x}\;\times \;N_{y}\;\times \;N_{z}\;\times \;L$, we build a high dimensional tensor ${\tilde{T}}_{p}\in \;C^{N\;\times \;K\;\times \;L}$ of K-1 similar 3D+L patches, with $N=N_{x}\;\times \;N_{y}\;\times \;N_{z}$ (see Figure 1, “unfolding” and “tensor stacking”). A fixed local window is used for the patch search, whereas the contrast signature remains unchanged. Along this line, the proposed reconstruction can exploit as much of the contrast and spatial correlations as possible.
Step 2 **–** The tensor ${\tilde{T}}_{p}$ exhibits a strong low multilinear rank structure and can therefore be compressed into a tensor of smaller size (i.e., the core tensor) through tensor decomposition (see Supporting Information Table S1 and Figure 1, “High‐Order Tensor Decomposition”). The dominant components of the core tensor can be extracted by computing a complex‐valued higher‐order singular value decomposition (HOSVD)^36^, ^37^ and by only keeping the largest (given by the thresholding parameter $\frac{2\lambda_{p}}{\mu }$) multilinear singular vectors and high‐order singular values. This step effectively acts as a high‐order denoising process where the small discarded coefficients mainly reflect contributions from noise and noise‐like artifacts.
Step 3 **–** The denoised tensor $T_{p}$ is then rearranged to form the denoised patches. Steps 1–3 are repeated over all patches in the image in a sliding window fashion. Because a single patch might belong to several groups in step 1, the final denoised multi‐contrast complex‐valued images T are obtained by averaging (Figure 1, “Aggregation”) the different estimates.

The solution T to this optimization problem is a denoised version of $\tilde{T}$ that is incorporated in the optimization 1 as prior knowledge, as described before. The Lagrangian multiplier b is then updated and optimizations 1 and 2 are processed iteratively to improve the quality of the reconstructed images. In the spirit of reproducible research, codes and examples for the proposed HD‐PROST technique are made available at http://www.kclcardiacmr.com/downloads/.

The generalized reconstruction framework described before considers 2D or 3D Cartesian multi‐contrast acquisitions (as the 3D undersampled Cartesian multi MT‐weighted acquisitions considered in this study). Slight modifications in the reconstruction process are required for the accelerated non‐Cartesian 2D MRF application considered in this study and will be described in the next section.

### 2.2 HD‐PROST for accelerated 2D radial parameter mapping with MRF

MRF^1^ is a novel quantitative MRI approach that allows the simultaneous acquisition of multi‐parametric maps (e.g., T_1_, T_2_, M_0_) in a single efficient scan. Conventional MRF sequences acquire in the order of thousands or more highly undersampled time point images by pseudo‐randomly collecting the MR data in a continuous fashion with time‐varying acquisition parameters (e.g., repetition time, flip angle). The spatial and temporal incoherencies provide a unique signal evolution (or fingerprint) for each tissue. These unique fingerprints can be matched, through pattern matching, to a pre‐generated MRF dictionary representative of the MRF sequence, and whose atoms are composed of simulated signal evolution curves. This matching process is performed on a voxel‐by‐voxel basis to identify the underlying tissue properties and generate quantitative parameter maps. The highly undersampled pseudo‐random MRF acquisition results in a high level of noise and aliasing in the reconstructed time point images. Several iterative techniques have been recently proposed to improve the reconstruction quality of each time point image.^38^, ^39^, ^40^, ^41^, ^42^ Zhao et al^38^ proposed to enforce low‐rank and subspace modeling in the temporal dimension to reconstruct high‐quality time point images. Assländer et al^39^ recently introduced a low‐rank ADMM reconstruction technique to temporally compress the time point images, resulting in a reduced number of singular value images. The reconstruction of the temporally compressed images is faster and better posed than reconstructing each time point image separately.^39^ This temporal compression operator $U_{r}$ is obtained through compression of the MRF dictionary at an appropriate rank r. Because of the multi‐contrast nature of MRF, HD‐PROST can be used to explicitly exploit the local, non‐local and contrast information of the temporally compressed images by integrating the compression operator into the encoding operator in Equation 3 as follows

(6) $$E_{\mathrm{MRF}}=AU_{r}FS$$

## 3 METHODS

The proposed HD‐PROST reconstruction was evaluated on accelerated radial 2D MRF phantom and in vivo brain acquisitions, and on accelerated Cartesian 3D magnetization transfer imaging with varying MT‐weighting in in vivo brain data. The 2 applications are described in detail below along with imaging and reconstruction parameters. Written informed consent was obtained from all subjects before undergoing MRI scans and the study was approved by the Institutional Review Board.

### 3.1 Accelerated 2D magnetic resonance fingerprinting

MRF acquisitions were performed on a 1.5 T Ingenia MR system (Philips, Best, the Netherlands) equipped with a 15‐element head coil.

#### 3.1.1 Phantom and in vivo experiments

A 2D MRF acquisition was performed on a standardized (T1MES) T_1_/T_2_ phantom containing 9 agarose‐based tubes with different T_1_ and T_2_ combinations (range, T_1_: 255 ms to 1489 ms, T_2_: 44 ms to 243 ms).^43^ Relevant scan parameters included: balanced steady‐state free precession radial sequence, TE = 2 ms, fixed TR = 4.4 ms, FOV = 160 × 160 mm^2^, in‐plane resolution = 1 × 1 mm^2^, slice thickness = 8 mm, bandwidth = 723.4 Hz/pixel. Only 1 radial spoke was acquired at each time point (resulting in an acceleration factor of ~251 with respect to a fully sampled radial acquisition). A total of 2000 time points were acquired in 10 s. A flip angle (FA) pattern similar to the one proposed in Assländer et al^44^ for optimized T_1_/T_2_ mapping was used and is shown in Supporting Information Figure S1. This RF pattern, which has been shown to be optimal in a Cramér‐Rao lower bound sense, consists of intrinsic repetitive loops that offers the advantage to lengthen the scan time by simple concatenation. The experiments consisted of undersampling the acquired data by keeping only $\left[1:n\right]$ k‐space radial spokes, with $n=\left[400:100:2000\right]$, resulting in scan time reductions up to a factor of 5 with respect to the 2000 time points sequence.

Reference T_1_ and T_2_ times for each vial were obtained from gold standard spin echo (SE) acquisitions. For T_1_ values, an inversion‐recovery SE (IRSE) sequence was used with 8 inversion times from 25 ms to 3200 ms with TR = 10s, TE = 14.75 ms. For T_2_ values, the SE sequence was performed with 8 TEs from 10 ms to 640 ms. T_1_ and T_2_ values were obtained by mono‐exponential curve fitting.

Single‐slice 2D MRF brain data were acquired in 5 healthy subjects (4 men, mean age: 32 years; range: 28–37 years) using the same scan parameters as in the phantom experiments.

#### 3.1.2 Image reconstruction

For both phantom and in vivo 2D MRF experiments, data was temporally compressed with r=10, leading to only 10 singular value images to reconstruct (i.e., in this study, L=10 and $M_{z}=1$).

HD‐PROST reconstruction was implemented using the algorithm described in Supporting Information Table S2 and performed offline on a workstation with a 16‐core Dual Intel Xeon Processor (23 GHz, 256 GB RAM). The joint MR reconstruction step (optimization 1) was implemented in MATLAB (v7.1, The MathWorks, Natick, MA) and the multi‐contrast patch‐based denoising step (optimization 2) in C. Coil sensitivity maps were estimated using the eigenvalue‐based approach ESPIRiT.^45^

The encoding operator $E_{\mathrm{MRF}}$ was implemented using the nonuniform fast Fourier transform.^46^ The tolerance of the conjugate gradient was set to $CG_{\mathrm{eps}}=1e^{-4}$ and a maximum number of $CG_{\mathrm{iter}}=15$ iterations was chosen as stopping criterion. The regularization parameter μ, which balances the contribution of the prior term (obtained at the end of optimization 2) and the data fidelity term, was set to $5e^{-3}$.

The proposed high‐order patch‐based denoising strategy was implemented as described in Supporting Information Table S1. The performance of the proposed strategy relies on the optimal selection of several parameters. The patch size, which controls the degree of local image features, was set to N=7×7. We set the search window radius around each pixel to 20 and restricted the number of similar patches selected to K=20 to form a third‐order tensor $T_{p}$ of size 49×20×10. The $l_{2}$ distance was chosen as measure of patch similarity and was defined as $d\left(patch_{\mathrm{ref}},patch_{j}\right)=\;\;{\left\|patch_{\mathrm{ref}}-patch_{j}\right\|}_{2}$ for j=1,…,K-1. To save computational complexity, a sliding‐window approach was performed with a patch offset of 3 pixels at each image dimension. The performance of HD‐PROST was assessed on several data sets (not reported here) by comparing the quality of the reconstructions with several regularization parameters λ (the same λ was used for all patches: $\lambda_{p}=\lambda$ for all *p*). The optimal value was shown to be proportional to the number of MRF measurements and was set to $\;\lambda =-1e^{-3}\;\times \;n\;+\;0.4$ for each decomposition, with n being the number of MRF radial spokes. The joint MR reconstruction and denoising steps were iteratively interleaved and the reconstruction was terminated after 5 ADMM iterations.

The proposed HD‐PROST reconstruction for 2D MRF was compared to the low‐rank inversion (LRI) reconstruction^24^, ^38^ with r=10 and using 10 conjugate gradient iterations, which were seen to be enough for convergence.

#### 3.1.3 Dictionary generation and pattern recognition

The MRF dictionary was generated using the extended phase graphs (EPG) formalism.^47^ The dictionary was calculated for a T_1_ in the range of ($\left[50:10:1400,\;1430:30:1600,\;1700:100:2200,\;2400:200:3000\right]$ ms) and T_2_ in the range of ($\left[5:2:80,\;85:5:150,\;160:10:300,\;330:30:600\right]$ ms). Slice profile was simulated for each RF pulse using 51 isochromats distributed along the slice selection direction and was included in the dictionary generation to correct for profile imperfections.^48^ Template‐matching between fingerprints and dictionary were performed using the inner product as in Ma et al.^1^

### 3.2 Accelerated 3D multi‐contrast magnetization transfer imaging

#### 3.2.1 Acquisition

A 3D accelerated MTC experiment was performed to evaluate the proposed HD‐PROST reconstruction on 3D Cartesian acquisitions with multiple MT‐weighted images. In vivo brain acquisitions were performed on 3 healthy subjects (1 man, age range: 24–30 y) on a 1.5 T MR scanner (Magnetom Aera, Siemens Healthcare, Erlangen, Germany) equipped with a 20‐channel head coil. Acquisitions consisted of 1 reference image without magnetization preparation and 5 images with different MT preparations (i.e., in this study, L=6 and $M_{z}>1$).

A prototype 3D Cartesian variable‐density trajectory was integrated in the sequence to allow for fast acquisition of multiple MT‐weighted images. The Cartesian trajectory with spiral profile order^33^, ^49^ samples the $k_{y}$‐$k_{z}$ phase‐encoding plane following approximate spiral interleaves on the Cartesian grid with variable density along each spiral arm and with 2 successive spiral interleaves being rotated by the golden ratio. A golden angle rotation between different contrast acquisitions was incorporated here (shifted VD‐CASPR) to introduce incoherently distributed aliasing artifacts along the contrast dimension and noise‐like artifacts in the spatial dimension, which is beneficial from a CS and low‐rank point of view.^50^

The MT weighting was achieved by applying a train of sinc‐shaped, off‐resonance RF pulses before image acquisition with the following parameters: MT off‐resonance frequency (ΔF) = 3 kHz, 20 MT pulse repetitions, MT bandwidth = 401 Hz/pixel. Relevant scan parameters included: 3D gradient echo sequence, axial orientation, FOV = 230 × 230 × 160 mm^3^, nominal resolution 1 × 1 × 2 mm^3^, FA = 15°, TE = 1.78 ms, TR = 4.06 ms, receiver bandwidth = 925 Hz/pixel, 32 readouts per spiral interleave. Six measurements were acquired with different MT pulse flip angles ($\alpha_{\mathrm{MT}}=\left[0^{\circ },\;160^{\circ },\;320^{\circ },\;480^{\circ },\;640^{\circ },\;800^{\circ }\right]$) with a 5‐s pause between them. Acquisitions were performed with an acceleration factor of 6.5‐fold for each weighted image. The total scan time to acquire the 6 measurements was 13:18 [min:s]. A fully sampled acquisition of the 6 measurements at this resolution would take more than 1 h. Therefore, for comparison purposes, an additional fully sampled acquisition was performed only for the reference image ($\alpha_{\mathrm{MT}}=0^{\circ }$). The total scan time for this single‐contrast fully sampled acquisition was 12:57 [min:s].

#### 3.2.2 Reconstruction

The following parameters were used for the 3D multi‐MT reconstruction: patch size N=7×7×7, search window = 20×20×20, number of similar 3D patches selected K=30, patch offset = 3, ADMM iterations = 5, $CG_{\mathrm{eps}}=1e^{-7}$, $CG_{\mathrm{iter}}=10$. The threshold parameters λ and μ were empirically set to 0.1 and $5e^{-3}$, respectively. Coil sensitivity maps were estimated from the fully sampled k‐space center using the eigenvalue‐based approach ESPIRiT.

The proposed HD‐PROST reconstruction was compared with 2 well‐established state‐of‐the‐art reconstruction techniques. The first technique is LLR, proposed by T. Zhang et al^26^ for accelerating MR parameter mapping. LLR exploits the redundancy in the contrast dimension on local image regions in an iterative low‐rank framework. LLR was implemented using our ADMM framework by replacing the patch‐based denoising step by the low‐rank thresholding. This allows for fair comparisons because the same optimization was used, and only the manner in which the denoising is performed was modified. The rank threshold $\lambda_{\mathrm{LLR}}$ was fixed and set to 5% of the highest singular value. Because the acquired MT‐weighted data was fully sampled in the read‐out direction, the MR reconstruction step was accelerated for both LLR and HD‐PROST reconstructions by computing a 1D inverse FFT and considering multiple separable 2D reconstruction problems independently.

The second technique is an iterative CS reconstruction with spatial Wavelet sparsity constraint as described in Lustig et al^12^ and implemented in the BART toolbox.^51^ CS reconstruction was performed for each contrast independently. The regularization parameter $\lambda_{\mathrm{CS}}$ was optimized experimentally and set to 0.01. Visual assessment was performed between the different techniques and the fully sampled acquisition.

## 4 RESULTS

### 4.1 Accelerated 2D magnetic resonance fingerprinting

#### 4.1.1 Phantom study

**Figure 2:**
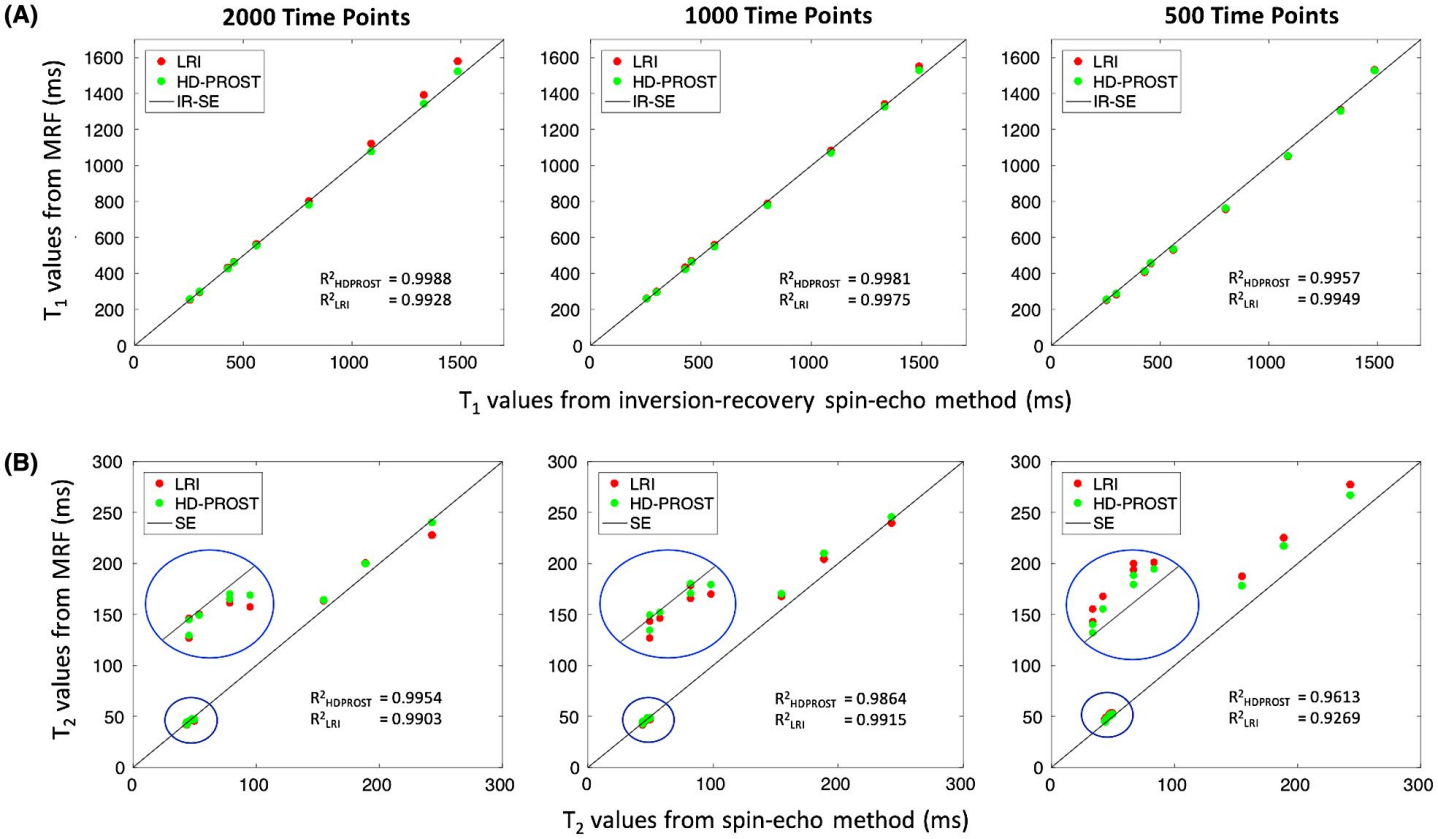
Phantom results for the 2D accelerated MRF using low‐rank inversion (LRI) and the proposed HD‐PROST reconstructions. Plots are comparing the mean T_1_ (A) and T_2_ (B) values derived from 2000, 1000, and 500 time points, with conventional inversion‐recovery spin‐echo (IRSE) and spin‐echo (SE) acquisitions (identity lines). T_1_ and T_2_ accuracies are preserved with the 2 strategies, with a slight bias observed for long T_2_s at high accelerations for both methods. The mean values were obtained from ROIs drawn around each phantom vial. Abbreviations: LRI, low‐rank inversion; HD‐PROST, high‐dimensionality undersampled patch‐based reconstruction

**Figure 3:**
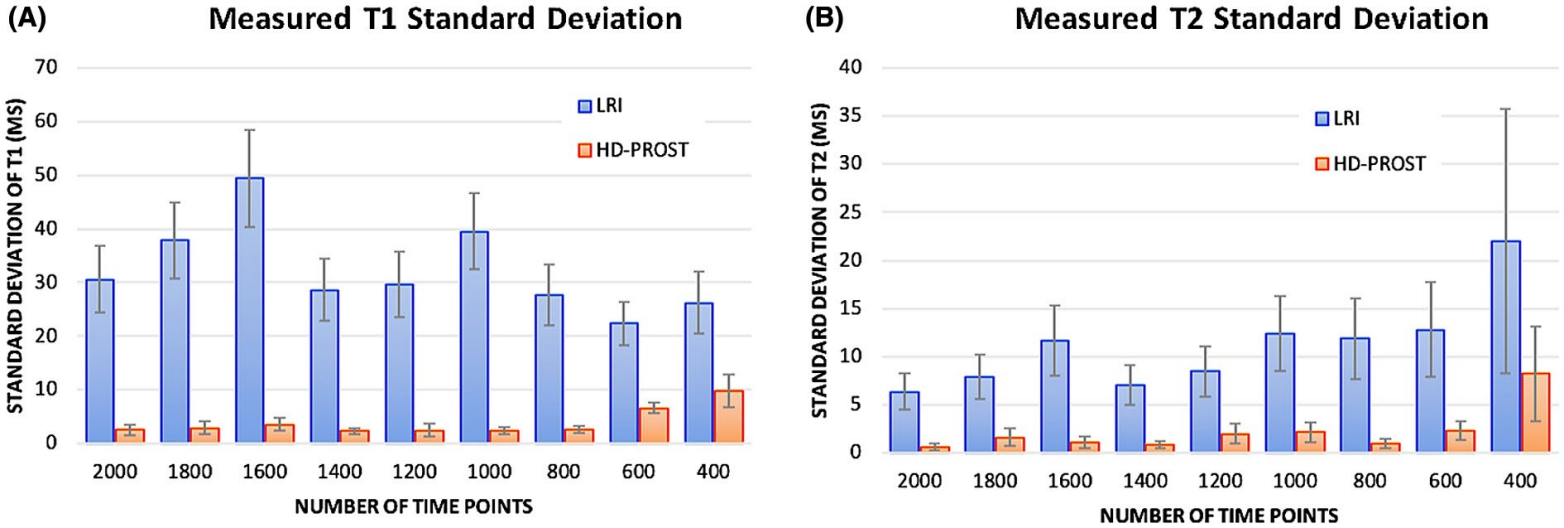
Standard deviations of T_1_ (A) and T_2_ (B) relaxation times for the phantom study are shown for LRI and HD‐PROST reconstructions for [400:200:2000] acquired time point images. The precision, as indicated by the SD, was considerably higher with the proposed HD‐PROST reconstruction, even for shorter acquisitions, whereas LRI resulted in systematic higher standard deviations. The SDs were obtained from ROIs drawn around each phantom vial. Abbreviations: LRI, low‐rank inversion; HD‐PROST, high‐dimensionality undersampled patch‐based reconstruction

Figure 2 shows T_1_ and T_2_ values for the 2D MRF phantom experiments with 2000, 1000, and 500 time points in comparison to the gold standard IRSE and SE acquisitions for both LRI and HD‐PROST reconstructions. T_1_ values obtained from both strategies were in good agreement with the IRSE acquisition even for reconstructions with 500 time points, with an excellent linear relationship with the reference T_1_ values (goodness‐to‐fit $R^{2}>0.98$). T_2_ accuracy was also preserved with the proposed reconstruction with a slight T_2_ degradation observed for long T_2_ values and high acceleration for both reconstructions. Figure 3 depicts the precision of T_1_ and T_2_ values, as characterized by the SD (aggregated based on the variance of each vial). An increase in precision was observed for both T_1_/T_2_ values using the proposed HD‐PROST reconstruction compared with LRI even for reconstructions with 500 time points, corresponding to 2.5s scan time. Corresponding T_1_ and T_2_ maps are shown in Supporting Information Figure S2. From the above analysis, it follows that 500 MRF time points or less might be sufficient and suitable for accurate and precise in vivo T_1_/T_2_ maps acquisitions in <2.5 s.

#### 4.1.2 In vivo study

**Figure 4:**
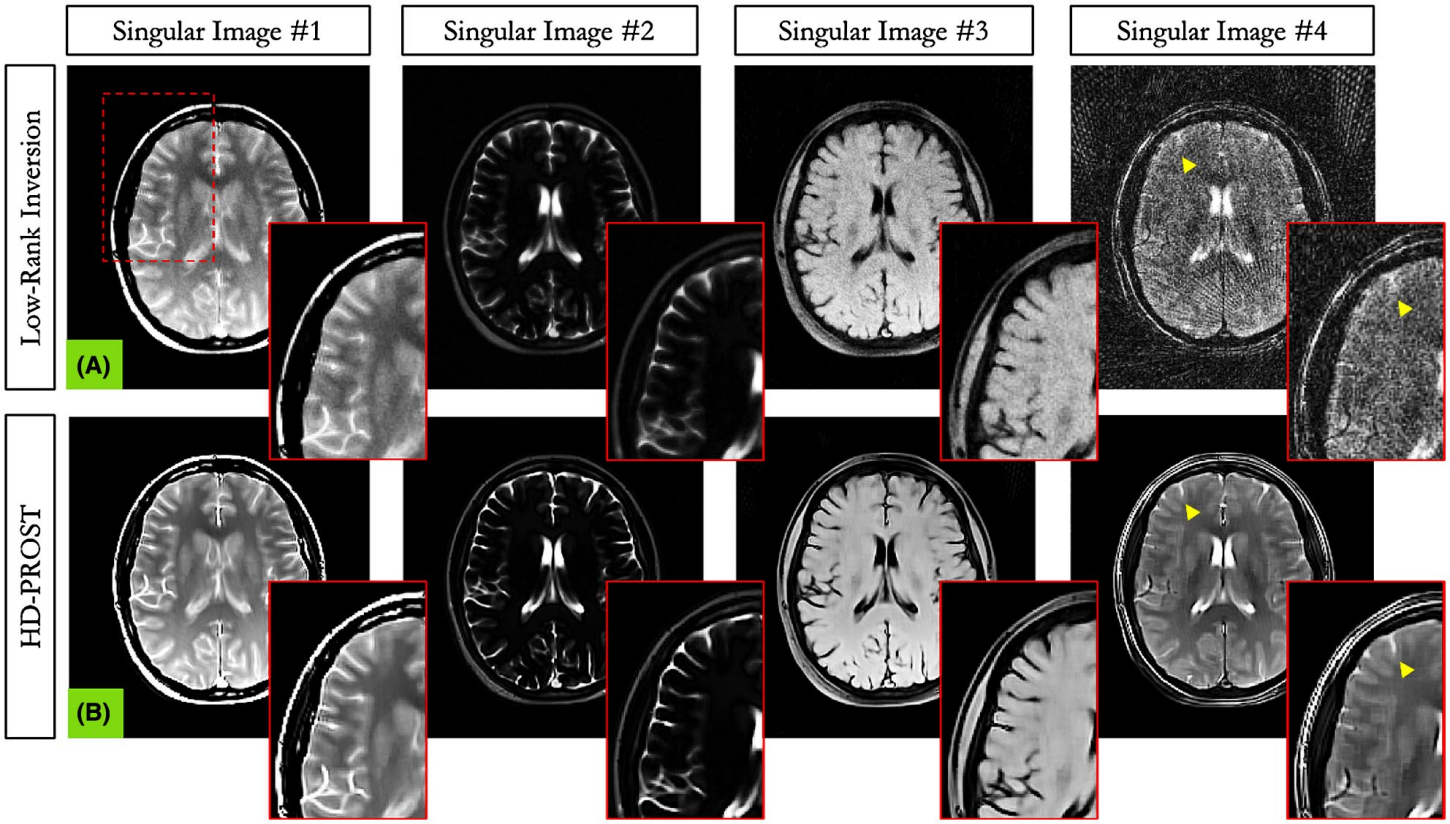
Reconstructed first 4 MRF singular images with low‐rank inversion (LRI) (A) and the proposed HD‐PROST (B) in in vivo brain experiments in a representative subject acquired with 1000 time points. A clear improvement in image quality and image sharpness can be observed on the HD‐PROST reconstruction with considerable reduction of noise and streaking artifacts, particularly for the last singular images

**Figure 5:**
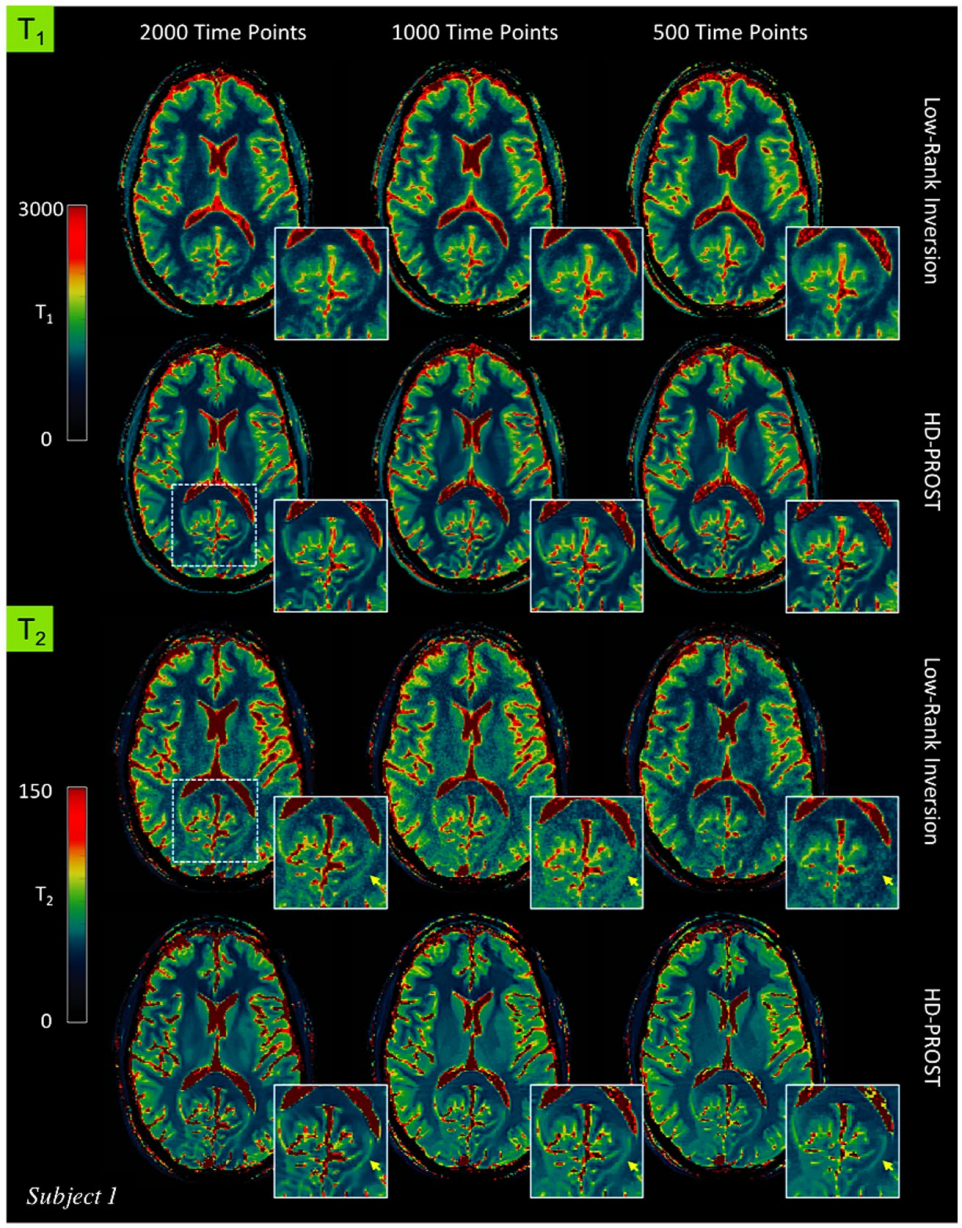
In vivo MRF‐derived quantitative T_1_ (top) and T_2_ (bottom) maps for subject 1 reconstructed with low‐rank inversion (LRI) MRF and the proposed HD‐PROST reconstruction with 2000, 1000, and 500 time points

**Figure 6:**
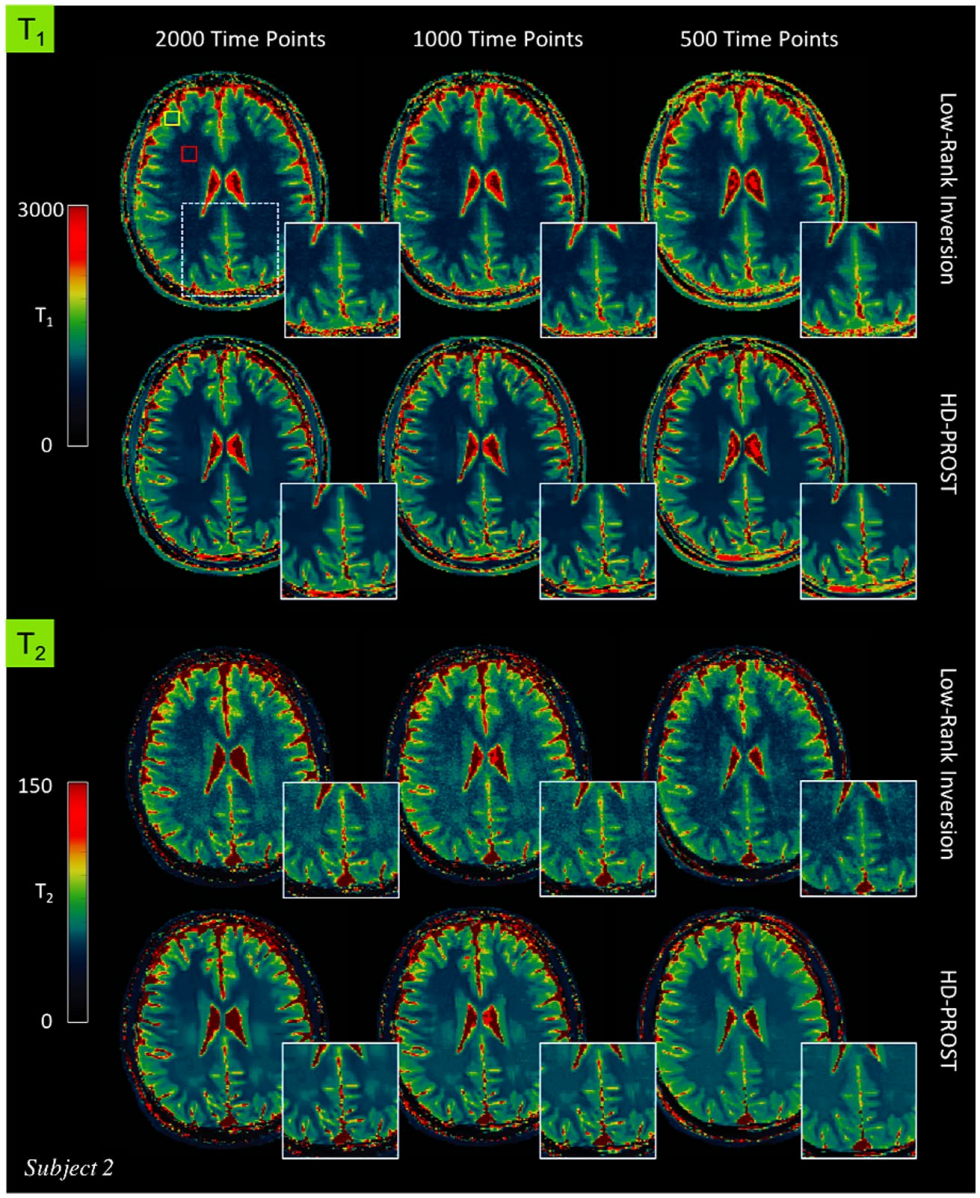
T_1_ (top) and T_2_ (bottom) maps for subject 2 reconstructed with low‐rank inversion (LRI) MRF and the proposed HD‐PROST reconstruction with 2000, 1000, and 500 time points. The yellow and red rectangles on the top‐left map indicate the regions of interest used to determine the T_1_ and T_2_ relaxation times (see Table 1)

Figure 4 depicts the first 4 2D MRF singular images from the reference LRI and the proposed HD‐PROST reconstruction for 1 representative subject reconstructed with 1000 time points. A clear superior image quality can be observed on the HD‐PROST singular images with a sharp and clear delineation of the brain structures. A high level of streaking artifacts and noise can be seen on the last singular value components (e.g., singular images 3 and 4) with LRI, whereas HD‐PROST not only produces images with considerably less noise but is also able to recover small structures that were lost below the noise level with LRI (Figure 4, yellow arrows). T_1_ and T_2_ maps are displayed in Figures 5 and 6 for 2 subjects and 3 different measurement lengths (2000, 1000, and 500 time points) for both LRI and HD‐PROST reconstructions.

The reconstructed maps from 1 additional subject are shown in Supporting Information Figure S3. A number of interesting observations can be made. Reducing the number of measurements tends to blur the T_1_ maps with LRI whereas the T_2_ maps suffer from noise amplification, showing an overall noisier appearance. Conversely, by enforcing low‐rank in the local, non‐local and contrast dimension, HD‐PROST reconstruction delivers higher image quality, recovering sharpness for T_1_ and reducing the noise for T_2_. The improvement is more pronounced for the 500 time points acquisition (2.5 s scan time). In vivo T_1_ and T_2_ relaxation times measured in regions of interest in the white and gray matters with LRI and the proposed HD‐PROST are shown in Table 1. Both reconstructions converged to very comparable values that are in good agreement with values obtained from the literature for T_1_. Moreover, the proposed HD‐PROST reconstruction tends to lower the SDs of T_1_ and T_2_ times, which is in accordance with the noise reduction seen in the quantitative maps. Note that the T_2_ relaxation times for both techniques are slightly biased and depart from the literature values. This may be partly explained by the fact that B_1_ imperfections^52^ as well as other sources of bias such as magnetization transfer^53^ and diffusion‐weighting^54^ were not considered in the proposed study. The average reconstruction time for 2D MRF with HD‐PROST was ~10 min per data set. Additional comparisons with single‐contrast PROST reconstruction (i.e., reconstructing each singular image independently) and with a global low‐rank tensor decomposition (in the spirit of cardiac multitasking)^28^, ^29^ are provided in Supporting Information Figure S4.

**Table 1:** T_1_ and T_2_ relaxation times at 1.5 T for LRI and the proposed HD‐PROST in regions of interest covering white and gray matters in the 5 healthy subjects (regions of interest are drawn in the maps in Figure 6)

	Number time points	T_1_ (ms)			T_2_ (ms)		
		LRI	HD‐PROST	Literature	LRI	HD‐PROST	Literature
White matter	2000	737 ± 61	743 ± 37		45 ± 5	45 ± 4	
	1000	718 ± 63	732 ± 36	608–756	47 ± 6	46 ± 4	54–81
	500	741 ± 64	746 ± 44		42 ± 4	45 ± 3	
Gray matter	2000	999 ± 117	992 ± 106		55 ± 6	54 ± 4	
	1000	988 ± 125	982 ± 108	998–1034	57 ± 6	56 ± 4	78–98
	500	1059 ± 151	1024 ± 128		52 ± 7	55 ± 4	

Abbreviations: LRI, low‐rank inversion; HD‐PROST, high‐dimensionality undersampled patch‐based reconstruction.

Values are shown for different MRF measurement lengths and compared with the corresponding literature values. Values are expressed as mean ± SD.

### 4.2 Accelerated 3D multi‐contrast magnetization transfer imaging

**Figure 7:**
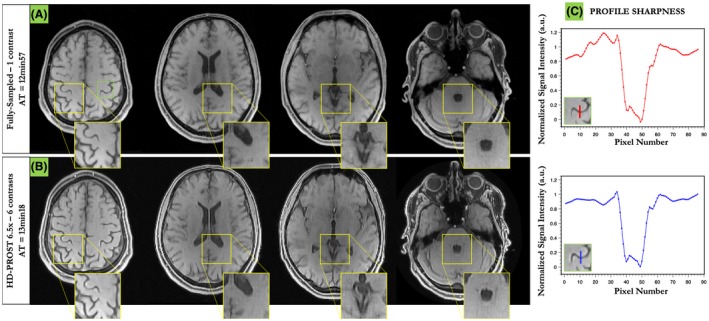
Three‐dimensional reconstruction of a MT‐weighted 6.5‐fold undersampled brain data in a healthy subject (subject 1). HD‐PROST reconstruction (B) is compared to the fully sampled acquisition (A) for the reference image only ($\alpha_{\mathrm{MT}}=0^{\circ }$). Line profiles going through a structure with sharp edges are shown in (C). HD‐PROST is able to recover high fidelity 3D images and retrieve sharp edges in agreement with the fully sampled acquisition. Six different undersampled MT‐weighted images were acquired in 13 min 18 s, whereas the fully sampled acquisition of a single contrast took 12 min 57 s

**Figure 8:**
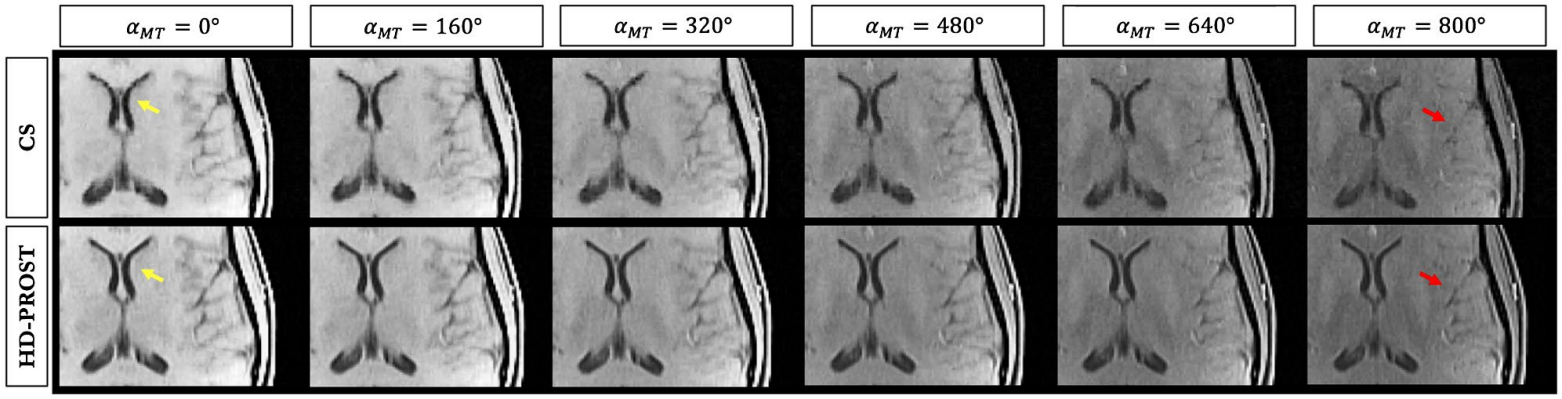
6.5‐fold accelerated 3D MT‐weighted images for 6 different contrasts from 1 representative subject (subject 1) reconstructed with compressed‐sensing (CS) and the proposed HD‐PROST reconstruction. Fine anatomic structures can be efficiently retrieved with HD‐PROST as shown by the arrows. See Supporting Information Figure S5 for the visualization of the whole axial images and Supporting Information Figure S6 for comparisons with zero‐filling and locally low‐rank reconstructions

Figure 7 depicts 4 axial slices obtained with HD‐PROST reconstruction of the 6.5‐fold undersampled 3D MT‐weighted images in a representative subject in comparison to the fully sampled acquisition. Only the reference image obtained with $\alpha_{\mathrm{MT}}=0^{\circ }$, is shown here. Similar image quality is observed between the 6.5‐fold accelerated HD‐PROST approach and the fully sampled scan. Line profiles going through a structure with sharp edges are shown in Figure 7C, showing excellent agreement between HD‐PROST and the fully sampled reference. Six different undersampled MT‐weighted images were acquired in 13 min 18s, whereas the fully sampled acquisition of a single contrast took 12 min 57 s. Figure 8 compares HD‐PROST to conventional CS reconstruction from a 6.5‐fold acceleration. Comparisons with zero‐filling and LLR reconstructions are provided in Supporting Information Figures S5 and S6. As expected, zero‐filling exhibits a low image quality with apparent aliasing artifacts and blurring. Exploiting contrast redundancy through local image regions with LLR improves the overall image quality and enables the recovery of small structures, particularly for low‐contrast images (e.g., $\alpha_{\mathrm{MT}}=800^{\circ }$), while the apparent noise is still large. By contrast, CS reconstruction with spatial regularization is able to recover images with reduced level of noise but fails to recover small structures for low contrast images (see Figure 8, red arrows). Enforcing multi‐dimensional low‐rank and capturing 3D information of local and non‐local 3D patches through the multiple MT‐weighted images with HD‐PROST allows to recover small structures and reduced the level of apparent noise, resulting in high image quality for all different contrasts. Reconstructions from 2 other subjects can be seen in Supporting Information Figures S7 and S8. The average computation time for 3D HD‐PROST reconstruction was ~27 min for all 6 contrasts in the acquisitions performed in this study.

## 5 DISCUSSION

HD‐PROST reconstruction enables accelerated acquisition of 2D or 3D multi‐contrast MR images by exploiting the high local and non‐local redundancies and the similarities between the multi‐contrast images through a high‐order low‐rank tensor approximation.

The proposed technique was applied to accelerated non‐Cartesian 2D MRF and accelerated Cartesian 3D MTC imaging to enable undersampling factors that go beyond the limit of traditional PI and CS reconstructions (i.e., ~2.5 s acquisition for 2D MRF and 6.5‐fold acceleration for 3D MTC), while removing residual aliasing artifacts. Phantom experiments in accelerated 2D MRF were carried out to investigate the impact of rapid acquisition (i.e., reduced number of time point images) on accuracy and precision of T_1_ and T_2_ relaxation times. High agreement with reference T_1_/T_2_ values was observed using HD‐PROST, even for high accelerations, with increased precision compared to conventional LRI reconstruction.

For in vivo MRF, streaking artifacts and noise amplification often propagated in the T_1_ maps with LRI reconstruction, whereas blurring was observed on the T_2_ maps for high acceleration factors. HD‐PROST achieved improved sharpness and reduced noise level in comparison to the low‐rank inversion reconstruction, especially for acquisitions with reduced number of time points. Nevertheless, a systemic underestimation of the T_2_ values, previously reported in MRF literature, was observed in the in vivo study. This finding may be partly explained by the fact that B_1_ imperfection,^52^ magnetization transfer,^53^ and diffusion‐weighting^54^ were not considered in this MRF study and could lead to inaccurate T_2_ measurements.

HD‐PROST has a modular design, which allows for its straightforward extension to 3D or n‐D imaging by simple patch vectorization. In line with the previous 2D MRF study, accelerated 3D MTC using HD‐PROST showed improved image quality over conventional CS and low‐rank reconstructions for an acceleration factor of 6.5, with visual quality comparable to the fully sampled acquisition. High denoising performance was achieved because of the existence of multiple MT‐weighted images of the same object with varying contrasts, leading to high redundancy that can be exploited by HD‐PROST. The pseudo‐random sampling, given by the proposed shifted VD‐CASPR, causes aliasing artifacts that spread incoherently in the contrast dimension and exhibits noise‐like perturbations at the image scale, providing an excellent basis for HD‐PROST reconstruction. This study was only performed on a small number of subjects and further evaluations on larger cohorts are needed. Nevertheless, this proof of concept suggests an opportunity for high‐resolution quantitative magnetization transfer imaging in a clinically feasible scan time.

The efficient multithreaded implementation of the high‐order patch‐based denoising allowed for fast image denoising of large data sets (e.g., in the order of 200 s for a 3D data set with a matrix size of 200×256×104×6). Further speedups could be achieved to reach clinically acceptable runtimes by implementing the joint MR optimization step on multiple GPUs^55^ and using coil compression algorithms.^56^

HD‐PROST imposes low‐rank in the complex domain and therefore captures the possible cross‐correlation observed between the real and imaginary components, allowing for accurate and faithful reconstruction of both phase and magnitude. Our framework makes use of ADMM to decouple the main optimization problem into 2 simpler sub‐problems that have straightforward solutions. Although most of the noise and undersampling artifacts can be efficiently removed after the first iteration, aliasing may still exist depending on the quality of the input images. This behavior mainly stems from the fact that corrupted images can negatively affect the block matching step, resulting in a sub‐optimal grouping. Therefore, several ADMM iterations (5 in this study) are needed to achieve good image quality reconstructions (see Supporting Information Figure S9).

The technique proposed in this article can potentially change conventional multi‐contrast imaging by making efficient use of the rich and redundant information available locally and temporally. Two applications were introduced in this study, nonetheless HD‐PROST stays generic and should be easily extendable to many MR applications where multiple contrasts are involved, such as conventional T_1_ and T_2_ mapping, perfusion imaging,^57^ 4D flow MRI,^58^ or low SNR applications such as arterial spin labeling.^59^

## 6 CONCLUSION

We present a new framework, termed HD‐PROST, for efficient reconstruction of undersampled multi‐channel multi‐contrast MR images. HD‐PROST aims at achieving high image quality by exploiting the high local and non‐local redundancies, and the similarities between the multi‐contrast images through a high‐dimensionality low‐rank tensor decomposition. HD‐PROST was validated in accelerated 2D MRF to generate precise T_1_ and T_2_ maps in ~2.5 s without affecting T_1_/T_2_ accuracy. For accelerated multiple 3D MT‐weighted acquisitions, HD‐PROST can recover high quality images, comparable to a fully sampled acquisition, in a clinically reasonable time frame. The straightforward, yet efficient, application of HD‐PROST to 2D and 3D multi‐contrast data sets provides several opportunities for future research, particularly in areas where high‐dimensionality is likely to increase in importance.

## Supporting information


**FIGURE S1** Variable flip angle pattern used in the accelerated 2D MRF study. This pattern was described in Assländer et al^44^
**FIGURE S2** T_1_ map (A) and T_2_ map (B) of the 2D MRF phantom acquisition. The quantitative values for all phantom tubes are reported in Figure 2. Abbreviations: LRI, low‐rank inversion; HD‐PROST, high‐dimensionality undersampled patch‐based reconstruction
**FIGURE S3** T_1_ (top) and T_2_ (bottom) maps for subject 3 reconstructed with low‐rank inversion MRF and the proposed HD‐PROST reconstruction with 2000, 1000, and 500 time points
**FIGURE S4** 2D MRF singular images (A) and corresponding T_1_ (top) and T_2_ (bottom) maps (B) for subject 2 reconstructed with low‐rank inversion (LRI), PROST (i.e., reconstructing each MRF singular image independently), global low‐rank tensor decomposition (global LR) and the proposed HD‐PROST reconstruction. The white rectangle on the top‐left map indicates the region of interest used to determine the T_1_ an T_2_ relaxation times. By exploiting local, non‐local, and contrast redundancies, the proposed HD‐PROST technique obtains better performance than the other techniques and reconstructs high‐quality T_1_ and T_2_ maps with great noise‐like artefacts reduction, contrast preservation, as well as sharpness enhancement, with T_1_ and T_2_ accuracies similar to the unregularized LRI reconstruction
**FIGURE S5** 6.5‐fold accelerated 3D MT‐weighted images for 6 different contrasts from subject 1 reconstructed with zero‐filling, locally low‐rank, compressed‐sensing, and the proposed HD‐PROST
**FIGURE S6** 6.5‐fold accelerated 3D MT‐weighted images for 6 different contrasts from 1 representative subject (subject 1) reconstructed with zero‐filling, locally low‐rank (LLR), compressed‐sensing (CS), and the proposed HD‐PROST. Fine anatomical structures can be efficiently retrieved with HD‐PROST as shown by the arrows. See Supporting Information Figure S5 for the visualization of the whole axial images. Note that slight residual motion can be observed on the sharp HD‐PROST reconstruction, which is lost in blurring on the compressed sensing reconstruction (because of regularization) and in the noise of LLR reconstruction
**FIGURE S7** 6.5‐fold accelerated 3D MT‐weighted images for 6 different contrasts from subject 2 reconstructed with zero‐filling, locally low‐rank, compressed‐sensing, and the proposed HD‐PROST
**FIGURE S8** Three‐dimensional reconstruction of a MT‐weighted 6.5‐fold undersampled brain data in a healthy subject (subject 3). HD‐PROST reconstruction is compared to the fully sampled acquisition for the reference image only ($\alpha_{\mathrm{MT}}=0^{\circ }$). Six different undersampled MT‐weighted images were acquired in 13 min 18 s, whereas the fully sampled acquisition of a single contrast took 12 min 57 s
**FIGURE S9** (A) plots of the residual norms for the primal, dual and Lagrangian variables as defined in the proposed HD‐PROST reconstruction against the number of ADMM iterations. (B) MRF T_1_ and T_2_ maps obtained with LRI and HD‐PROST are shown for different ADMM iterations (iterations 1, 5, and 10)
**TABLE S1** Algorithm I: high‐order tensor decomposition algorithm for HD‐PROST reconstruction
**TABLE S2** Algorithm II: high‐dimensionality undersampled patch‐based reconstruction (HD‐PROST)
