# Supplementary material for: High‐dimensionality undersampled patch‐based reconstruction (HD‐PROST) for accelerated multi‐contrast MRI
Source: Magn Reson Med. 2019 Mar 4;81(6):3705–19. doi: 10.1002/mrm.27694 (PMC6646908; doi:10.1002/mrm.27694)
Supplement: Supplementary file 1 — FIGURE S1 Variable flip angle pattern used in the accelerated 2D MRF study. This pattern was described in Assländer et al44 FIGURE S2 T1 map (A) and T2 map (B) of the 2D MRF phantom acquisition. The quantitative values for all phantom tubes are reported in Figure 2. Abbreviations: LRI, low‐rank inversion; HD‐PROST, high‐dimensionality undersampled patch‐based reconstruction FIGURE S3 T1 (top) and T2 (bottom) maps for subject 3 reconstructed with low‐rank inversion MRF and the proposed HD‐PROST reconstruction with 2000, 1000, and 500 time points FIGURE S4 2D MRF singular images (A) and corresponding T1 (top) and T2 (bottom) maps (B) for subject 2 reconstructed with low‐rank inversion (LRI), PROST (i.e., reconstructing each MRF singular image independently), global low‐rank tensor decomposition (global LR) and the proposed HD‐PROST reconstruction. The white rectangle on the top‐left map indicates the region of interest used to determine the T1 an T2 relaxation times. By exploiting local, non‐local, and contrast redundancies, the proposed HD‐PROST technique obtains better performance than the other techniques and reconstructs high‐quality T1 and T2 maps with great noise‐like artefacts reduction, contrast preservation, as well as sharpness enhancement, with T1 and T2 accuracies similar to the unregularized LRI reconstruction FIGURE S5 6.5‐fold accelerated 3D MT‐weighted images for 6 different contrasts from subject 1 reconstructed with zero‐filling, locally low‐rank, compressed‐sensing, and the proposed HD‐PROST FIGURE S6 6.5‐fold accelerated 3D MT‐weighted images for 6 different contrasts from 1 representative subject (subject 1) reconstructed with zero‐filling, locally low‐rank (LLR), compressed‐sensing (CS), and the proposed HD‐PROST. Fine anatomical structures can be efficiently retrieved with HD‐PROST as shown by the arrows. See Supporting Information Figure S5 for the visualization of the whole axial images. Note that slight residual motion can be observed on the [file MRM-81-3705-s001.docx]

**
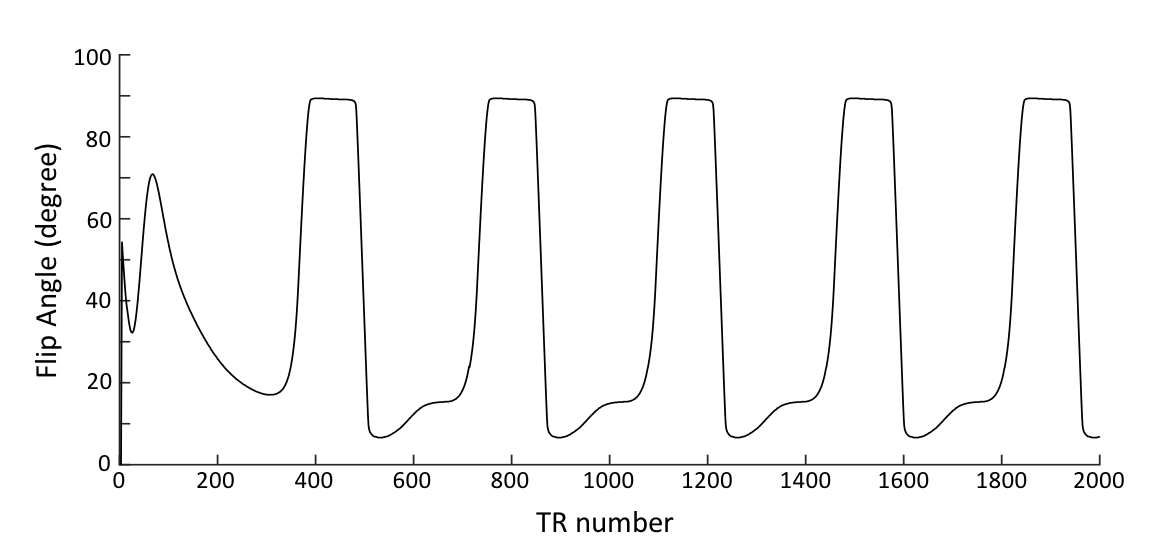
**

**Supporting Information** **Figure S1.** Variable flip angle pattern used in the accelerated 2D MRF study. This pattern was described in Assländer et al. (44)*.*

**
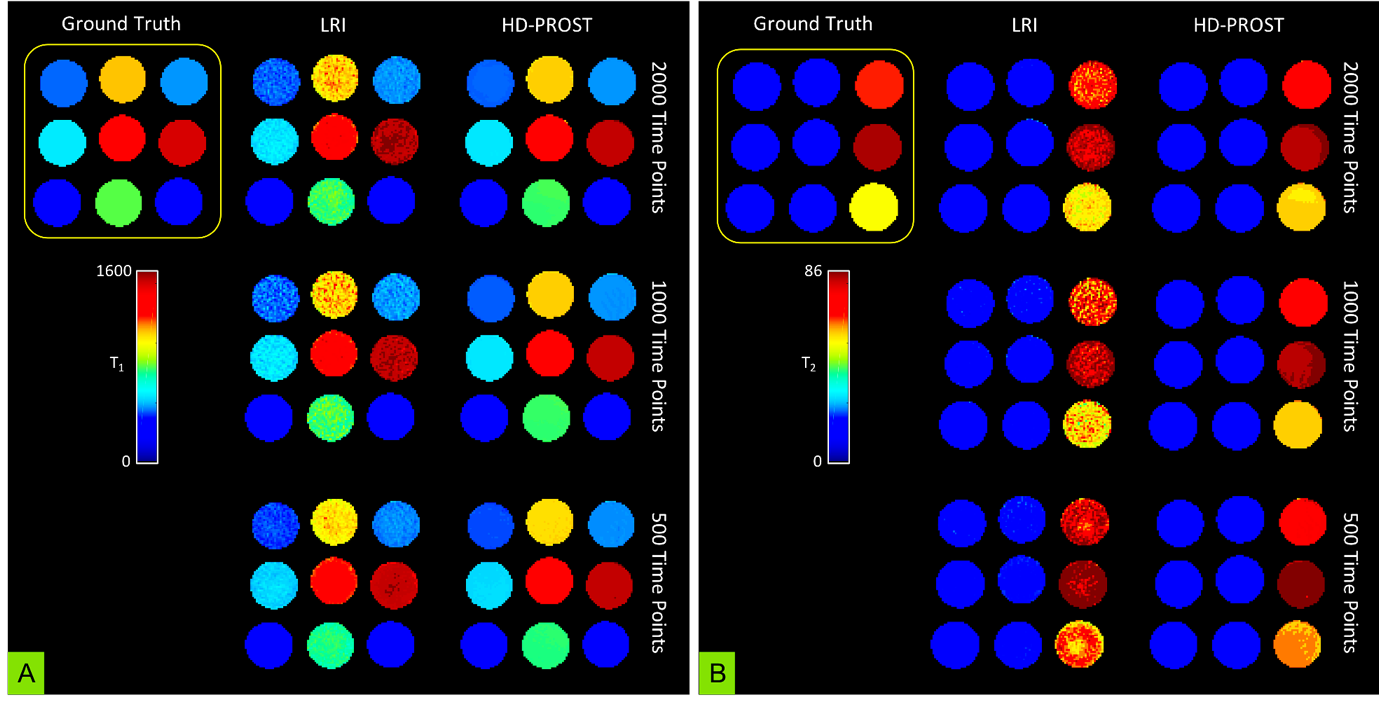
**

**Supporting Information** **Figure S2.** T_1_ map (A) and T_2_ map (B) of the 2D MRF phantom acquisition. The quantitative values for all phantom tubes are reported in Figure 2. Abbreviations – LRI: low-rank inversion, HD-PROST: high-dimensionality undersampled patch-based reconstruction.

**
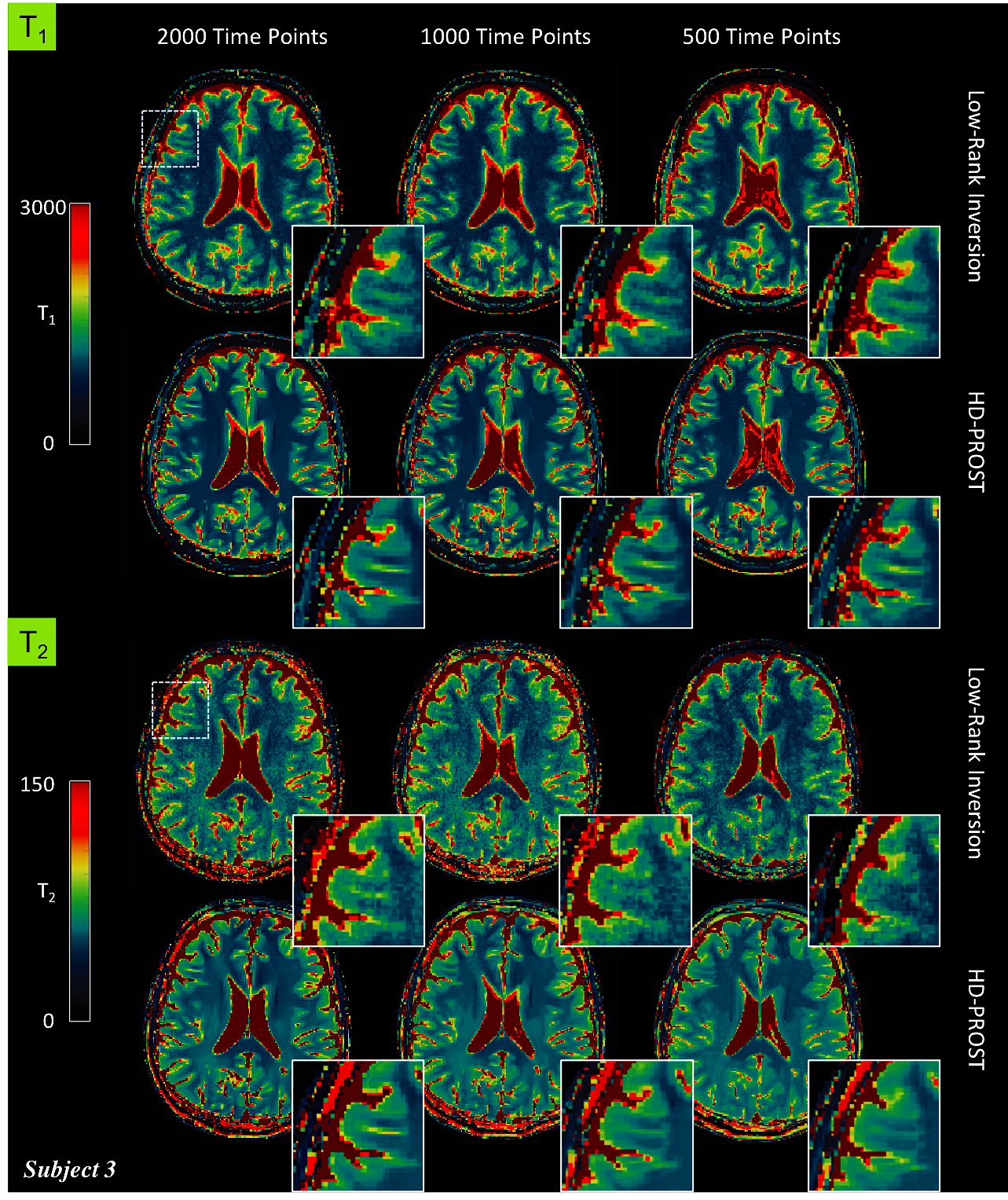
**

**Supporting Information** **Figure S3.** T_1_ (top) and T_2_ (bottom) maps for subject 3 reconstructed with low-rank inversion MRF and the proposed HD-PROST reconstruction with 2000, 1000 and 500 time-points.

**
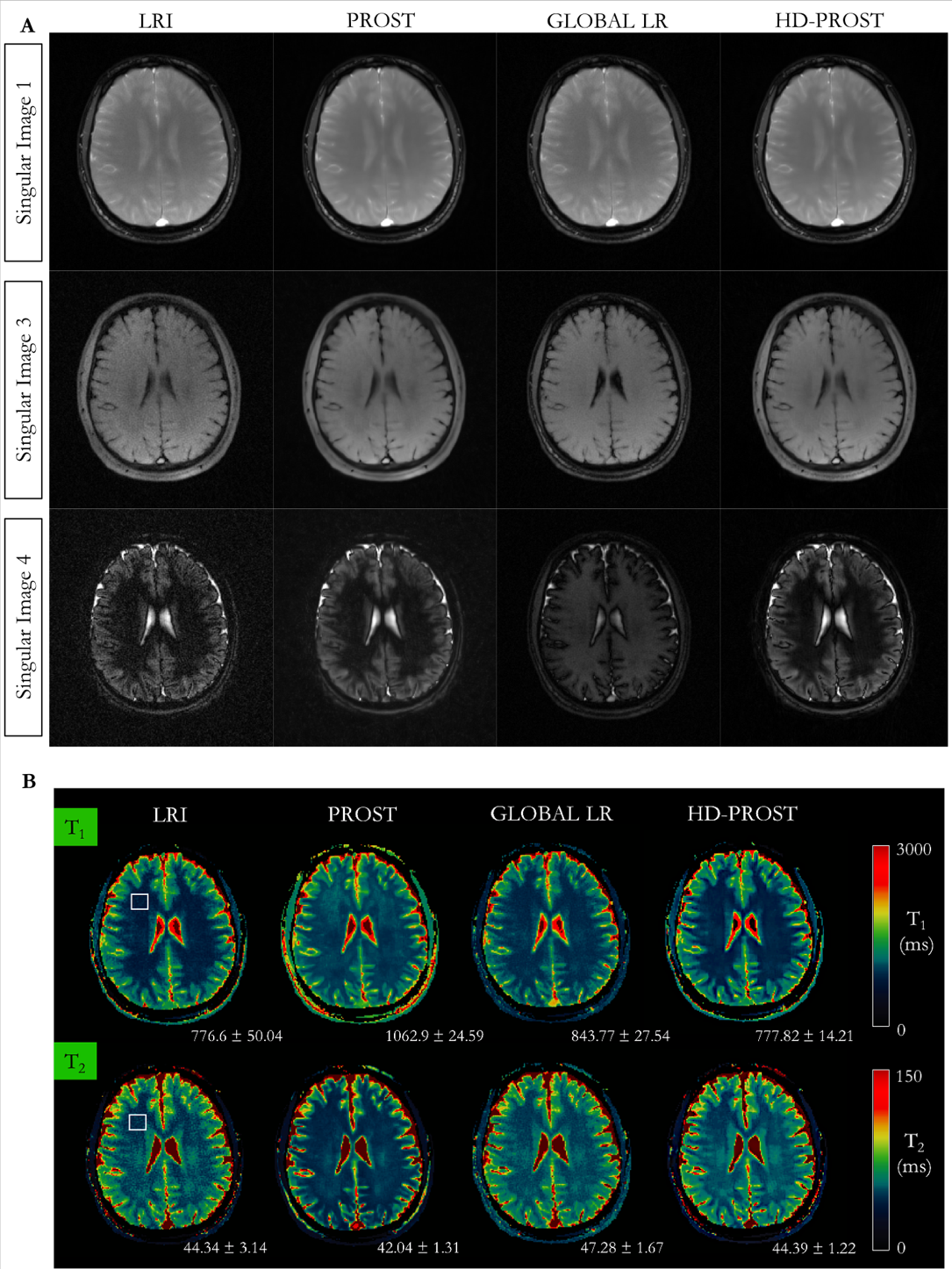
**

**Supporting Information** **Figure S4.** 2D MRF singular images (A) and corresponding T_1_ (top) and T_2_ (bottom) maps (B) for subject 2 reconstructed with low-rank inversion (LRI), PROST (i.e. reconstructing each MRF singular image independently), global low-rank tensor decomposition (global LR) and the proposed HD-PROST reconstruction. The white rectangle on the top-left map indicates the region of interest used to determine the T_1_ an T_2_ relaxation times. By exploiting local, non-local and contrast redundancies, the proposed HD-PROST technique obtains the best performance and reconstructs high-quality T_1_ and T_2_ maps with great noise-like artefacts reduction, contrast preservation, as well as sharpness enhancement, with T_1_ and T_2_ accuracies similar to the unregularized LRI reconstruction.

**
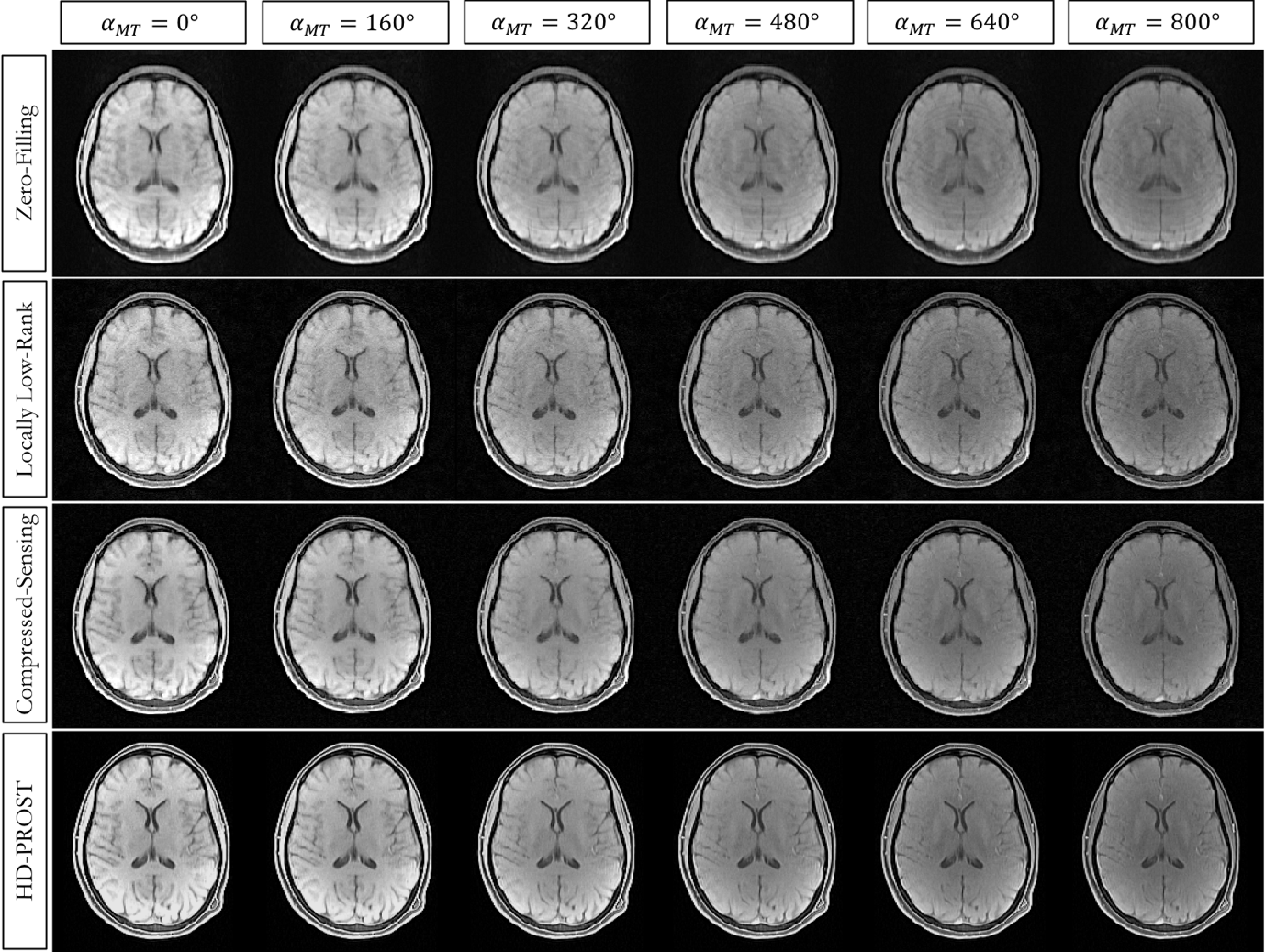
**

**Supporting Information** **Figure S5.** 6.5-fold accelerated 3D MT-weighted images for 6 different contrasts from subject 1 reconstructed with zero-filling, locally low-rank, compressed-sensing, and the proposed HD-PROST.

**
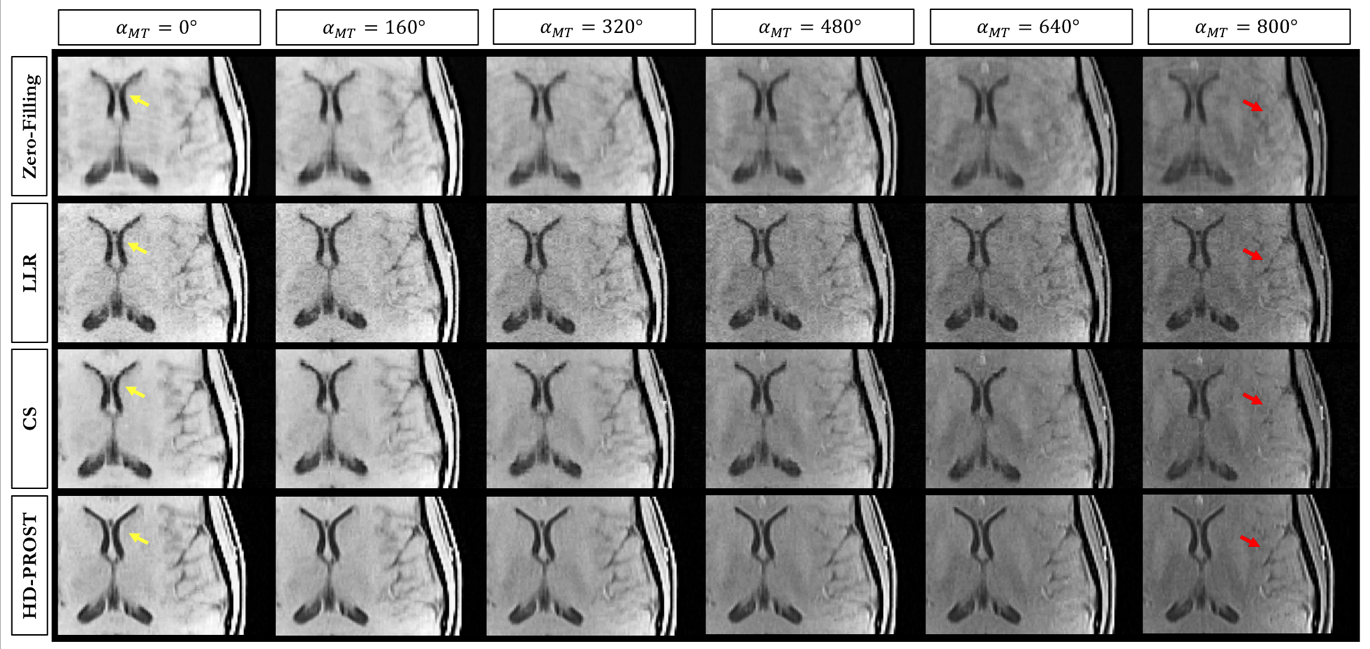
**

**Supporting Information** **Figure S6.** 6.5-fold accelerated 3D MT-weighted images for 6 different contrasts from one representative subject (subject 1) reconstructed with zero-filling, locally low-rank (LLR), compressed-sensing (CS), and the proposed HD-PROST. Fine anatomical structures can be efficiently retrieved with HD-PROST as shown by the arrows. See Supporting Information Figure S5 for the visualization of the whole axial images. Note that slight residual motion can be observed on the sharp HD-PROST reconstruction, which is lost in blurring on the compressed sensing reconstruction (due to regularization) and in the noise of LLR reconstruction.

**
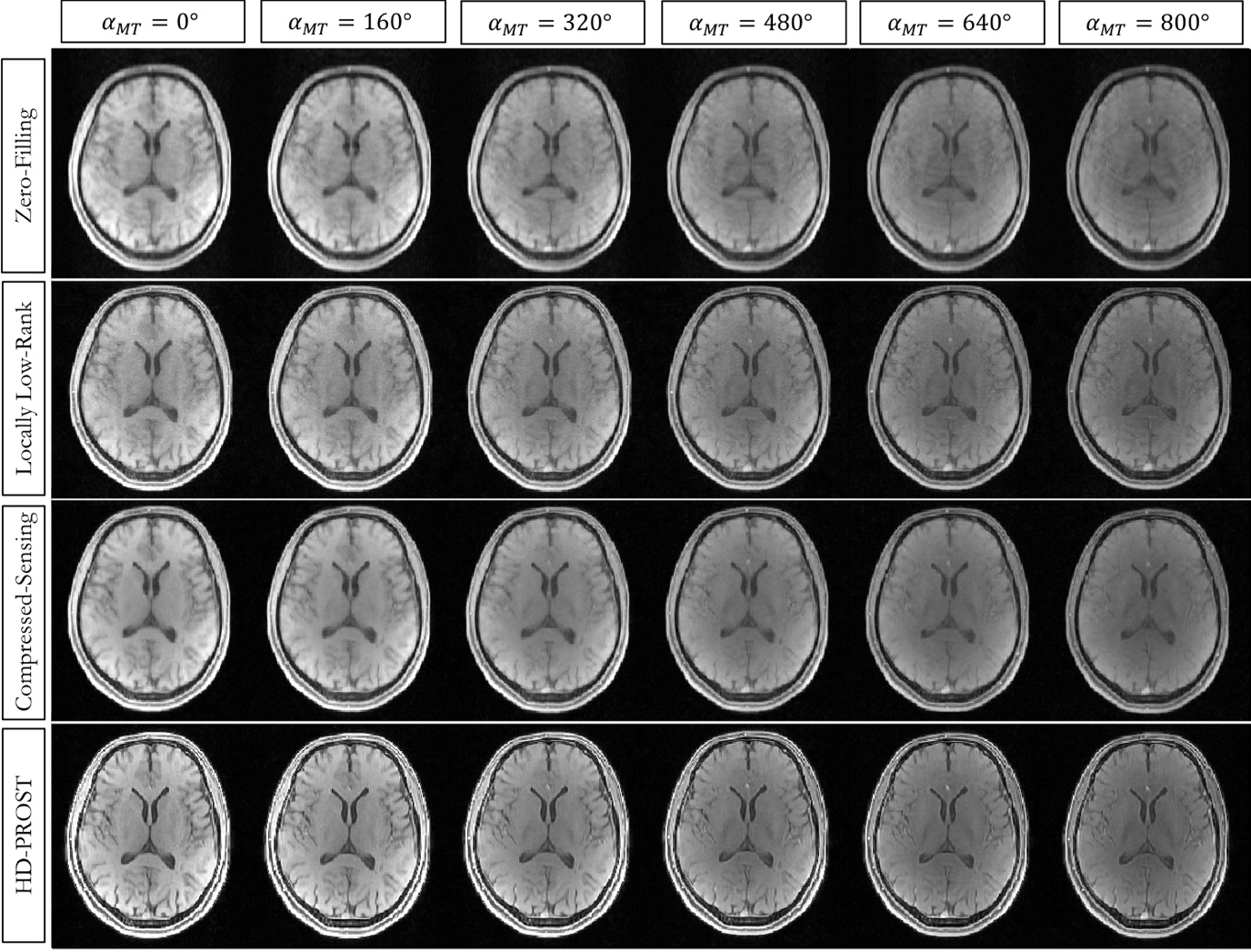
**

**Supporting Information** **Figure S7.** 6.5-fold accelerated 3D MT-weighted images for 6 different contrasts from subject 2 reconstructed with zero-filling, locally low-rank, compressed-sensing, and the proposed HD-PROST.

**
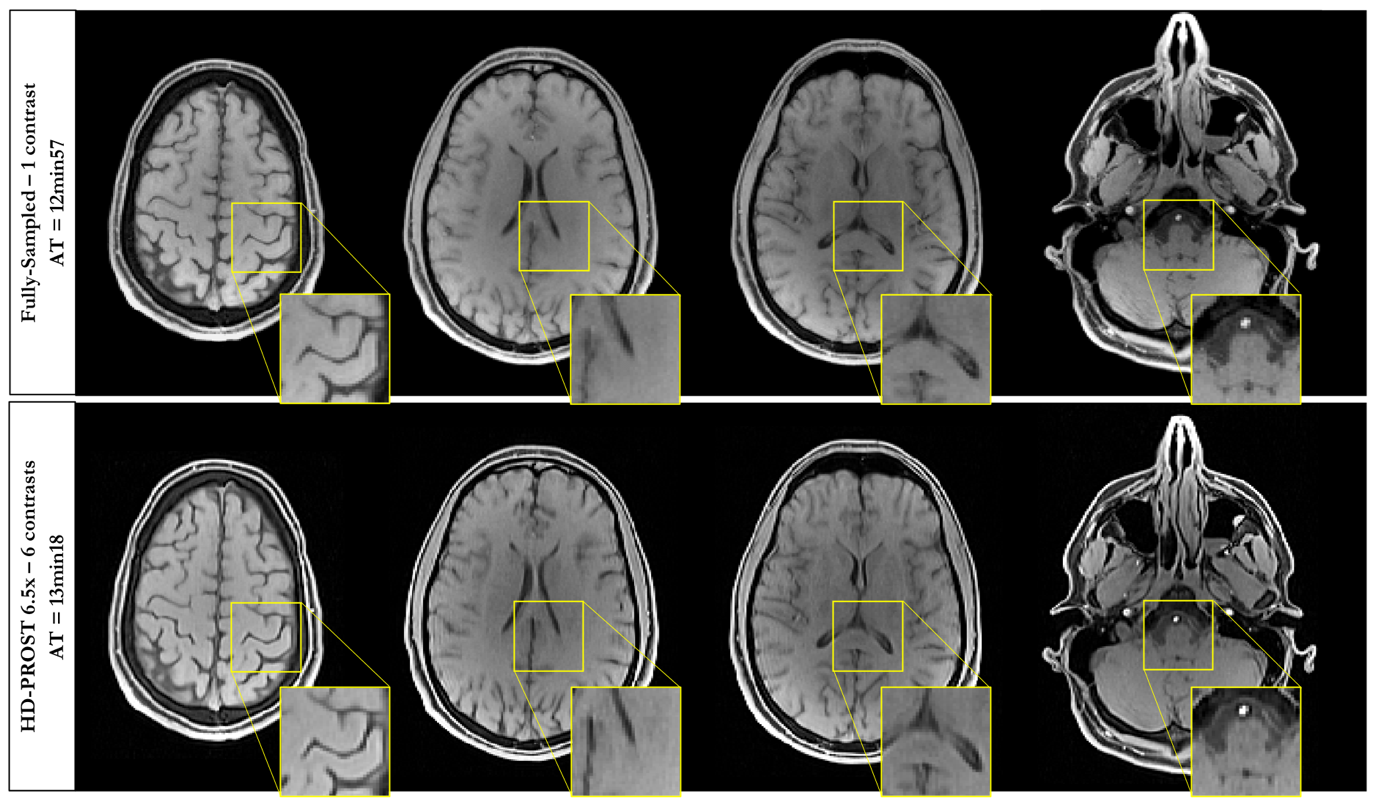
**

**Supporting Information** **Figure S8.** Three-dimensional reconstruction of a MT-weighted 6.5-fold undersampled brain data in a healthy subject (subject 3). HD-PROST reconstruction is compared to the fully-sampled acquisition for the reference image only ($\alpha_{MT}=0^{\circ}$). Six different undersampled MT-weighted images were acquired in 13min 18s, whereas the fully-sampled acquisition of a single contrast took 12min 57s.

**
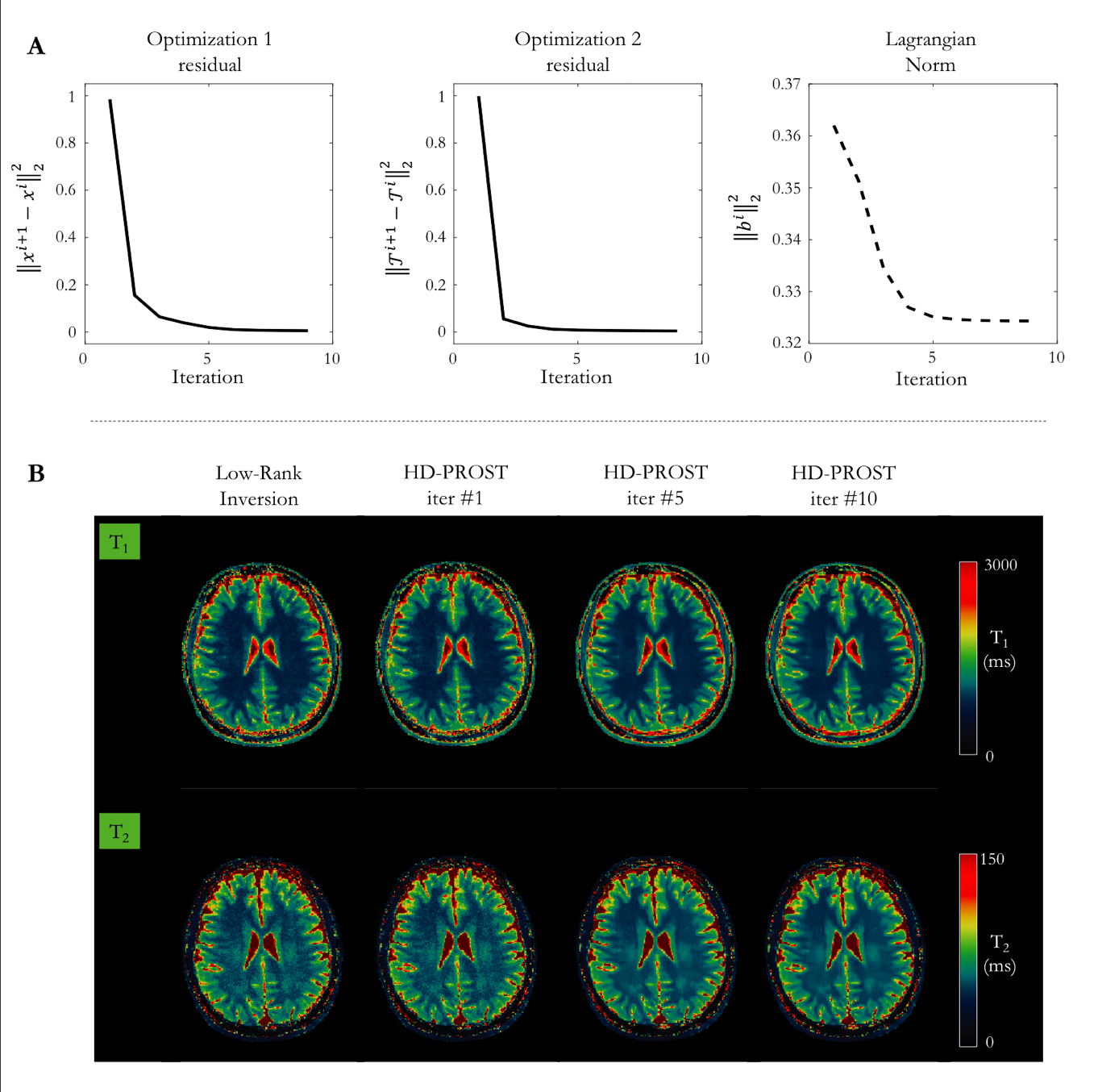
**

**Supporting Information** **Figure S9.** (A) plots of the residual norms for the primal, dual and Lagrangian variables as defined in the proposed HD-PROST reconstruction against the number of ADMM iterations. (B) MRF T_1_ and T_2_ maps obtained with LRI and HD-PROST are shown for different ADMM iterations (iterations 1, 5 and 10).

**Supporting Information Table S1**

ALGORITHM I

High-Order Tensor Decomposition Algorithm For HD-PROST Reconstruction

***INPUT:*** *data tensor $\mathcal{T}$ with dimensions $\left( N,K,L \right)$ and regularization parameter* $\lambda$

***ALGORITHM:***

1. *Unfold the tensor $\mathcal{T}$ along its single modes:*

$\mathcal{T}_{1}$*: which reshapes $\mathcal{T}$ into a $L\times\left( N .K \right)$ complex matrix*

$\mathcal{T}_{2}$*: which reshapes $\mathcal{T}$ into a $N\times\left( L .K \right)$ complex matrix*

$\mathcal{T}_{3}$*: which reshapes $\mathcal{T}$ into a $K\times\left( L .N \right)$ complex matrix*

1. *Compute the complex SVD of $\mathcal{T}_{n}$ and get the orthogonal matrices $U^{\left( 1 \right)}$, $U^{\left( 2 \right)}$, $U^{\left( 3 \right)}$ from the n^th^-mode signal subspace*
2. *Compute the complex core tensor $\mathcal{S}$ related by*

$$\mathcal{S=T}\times_{1} U_{(1)}^{H}\times_{2}U_{(2)}^{H}\times_{3}U_{(3)}^{H}$$

*Which is equivalent to its unfolding forms:*

$$\mathcal{S}_{n}=U_{(n)}^{H} .\mathcal{T}_{n}.\left[ U_{\left( i \right)} \bigotimes U_{\left( j \right)} \right] with 1\leq n\leq3 and i\neq j\neq n$$

*In which $\otimes$ denotes the Kronecker product*

1. *Compute the high-order singular values truncation (hard-thresholding):*

$$\mathcal{S}\left( \mathcal{S<}\lambda\right)=0$$

2. *Construct back the filtered tensor $\mathcal{T}_{(n)}^{den}$:*

$$\mathcal{T}_{(n)}^{den}=U_{(n)}\mathcal{. S .}\left[ U_{\left( i \right)} \bigotimes U_{\left( j \right)} \right]^{H} with 1\leq n\leq3 and i\neq j\neq n$$

***OUTPUT:*** *The denoised tensor $\mathcal{T}^{den}$ is obtained by folding*

**Supporting Information Table S2**

ALGORITHM II

High-Dimensionality Undersampled Patch-Based Reconstruction (HD-PROST)

***INPUT:*** *undersampled multi-channel multi-contrast images* $X$

*parameters $\lambda_{p}, \mu$, ADMM iterations* $ADMM_{iter}$

*Encoding operator E (coil sensitivities $S$, sampling mask $A$)*

*Compression operator $U_{r}$ (for MRF)*

***INITIALIZATION:***

*Solve optimization 1 (Eq. 4): Joint MR reconstruction without prior ($\mu=0$)*

*%* ***Output****:* $X^{\left( 0 \right)}$

***ALGORITHM:***

$$for i=1, \ldots,ADMM_{iter}$$

*Solve optimization 2 (Eq. 5): HOSVD-based denoising (see Algorithm I)*

*%* ***Outpu****t: denoised tensor* $\mathcal{T}^{\left( i \right)}$

*Solve optimization 1 (Eq. 4): Joint MR reconstruction with prior*

*%* ***Output****: reconstructed images* $X^{\left( i \right)}$

*Update Lagrangian multiplier:*

$b^{\left( i \right)}=b^{\left( i-1 \right)}+X^{\left( i \right)}-\mathcal{T}^{\left( i \right)}$

$$end for$$

***OUTPUT:*** *The multi-contrast images* $X$
